# Comprehensive review of dermatological and cosmeceutical manifestations of thermal water and future insights

**DOI:** 10.1007/s00484-025-02937-0

**Published:** 2025-05-27

**Authors:** Shamsa Kanwal, Enass Y. Osman, Imen Khiari

**Affiliations:** 1 Department of Medical, Oral and Biotechnological Sciences, University of Chieti – Pescara “G. d’Annunzio”, 66100 Chieti, Italy; 2 Department of Pharmacology and Toxicology, Tanta University, Tanta, Egypt; 3 Georesources Laboratory of Water Research and Technologies Center (CERTE), Technopole Borj Cedria, BP273 Soliman, Tunisia

**Keywords:** Thermal water, Balneotherapy, Therapeutic effects, Cosmetic effects, In vitro, In vivo, Skin diseases

## Abstract

Balneotherapy is a widely accepted and well-practiced treatment modality for dermatological diseases in many countries worldwide. The use of balneotherapy approaches for skin disease management is continuously increasing, but the mechanism by which thermal water affects the treatment of different dermatological conditions has not been fully explored. This comprehensive review seeks to consolidate scientific findings regarding the effects of thermal water in the treatment of different skin diseases, such as psoriasis, atopic dermatitis, acne, ichthyosis, and lichen planus, as well as its cosmeceutical effects, drawing from both in vitro and in vivo research. This review analyzed both in vitro and in vivo studies to validate the anti-inflammatory, immunomodulatory, and antioxidant potential of thermal water in dermatology. Thermal water exhibits these properties, making it beneficial for treating various skin conditions and enhancing cosmeceutical effects." Many studies have strongly demonstrated the therapeutic benefits of different thermal waters for the successful treatment of many skin diseases, and these materials have been incorporated into cosmetics for skin hydration and anti-aging and anti-wrinkle effects. The physicochemical and microbial characteristics of thermal water contribute to its therapeutic properties, with sulfurous water being particularly effective due to their favorable mineral composition and antioxidative effects.Although the cosmetic properties of thermal water are well described, further research is needed to identify specific mineral and microbial profiles for incorporation into cosmetic products and to integrate advanced technologies and approaches to increase the effectiveness of formulated products. **S**hould aim to clarify the underlying mechanisms of action of thermal waters, investigate optimal treatment protocols, and assess their long-term efficacy. Longitudinal clinical trials are also needed to assess the long-term benefits and safety of these treatments, paving the way for standardized protocols in dermatological care.

**Graphical Abstract:**

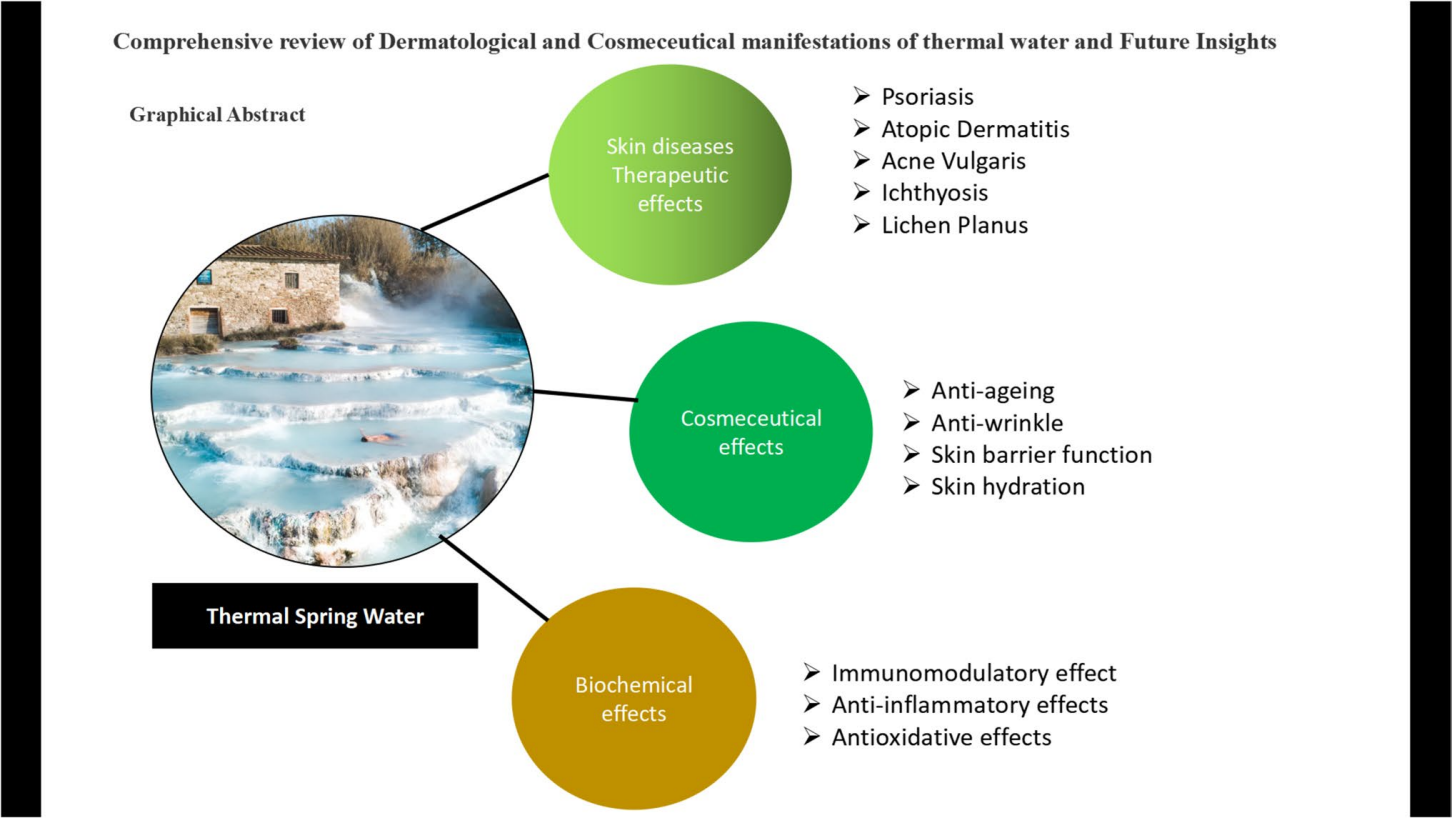

## Introduction

The skin is the largest organ of the body and has different functions, acting as a protective barrier against many pathogens and physiochemical and microbiological stressors. It also plays an important role in excretory, thermoregulatory, and sensory functions. Skin diseases are the most common and distressing life conditions of more than a million people globally. Numerous studies have reported that approximately 70% of the total world population suffers from skin diseases of varying severity. (Hay et al. 2014; Karimkhani et al. 2017; Sanclemente et al. 2017). The most prevalent skin diseases are induced by infectious agents such as viruses, bacteria, and fungi (Jain et al. 2016). Some common and chronic skin diseases are psoriasis, atopic dermatitis, vitiligo, itching, ichthyosis, acne vulgaris, and allergic contact dermatitis. The use of thermal water for managing different skin diseases is traditional treatment with least side effects and more reliability (Protano et al. 2024). In the last two decades, the curative effects of thermal water have been extensively explored, with studies showing that its physiochemical, microbiological, immunological, and mechanical properties play a significant role in treating various skin conditions Therefore, the thermal water used for the treatment of different skin conditions has specific physiological, mineral, and chemical profiles (Araujo et al. 2017). The most effective mineral profile for skin disease treatment reported thus far is thermal water rich in sulfur, carbonates, bicarbonates, calcium, sulfate, and silicon. Recently, thermal medicine has been well characterized as a branch of medicine in which the therapeutic and curative properties of water are used in different treatment regimens, including those for skin diseases (Cacciapuoti et al. 2020), rheumatoid diseases (Fioravanti et al. 2011), respiratory diseases (Viegas et al. 2019) and cardiovascular diseases (Oyama et al. 2013). The skin diseases most extensively treated with thermal water are psoriasis, atopic dermatitis, ichthyosis, acne vulgaris, rosacea, and lichen planus. Despite the successful treatment of these diseases, the exact biological mechanism underlying their effects has not yet been elucidated. The therapeutic effects of thermal water are believed to be driven by its effects on immunological and neuroendocrine functions..

Considering the significant curative, anti-irritant, anti-inflammatory, antioxidant, immunomodulatory, and skin regenerative properties of thermal water, it can be used as a whole as well as its active ingredients in different topical formulations as therapeutic agents and cosmetics is justified.

## Materials and methods

This comprehensive review explicitly analyzes the literature on the therapeutic and cosmetic effects of thermal water and highlights its potential beneficial properties. A systematic search was conducted in the PubMed and Google Scholar databases from January 2000 to September 2024 via the following search terms:"thermal water,""balneotherapy,""cosmetic effect,""balneotherapy,""spa therapy,""antioxidants,""oxidants/reactive oxygen species,""skin diseases,""in vitro studies,"and"in vivo studies"via OR and AND operators. Both the clinical and preclinical studies were targeted to obtain a comprehensive view. Studies were included if they targeted the problem under study, describing the cosmetic effects and skin disease treatment with thermal water. Studies not related to skin diseases or cosmetic or therapeutic effects were excluded. In total, 81 studies that met the inclusion criteria of our review were filtered out. For a systematic presentation of the literature review, the extracted data are organized in Tables 1, 2, 3 and 4 splitting on the basis of in vitro and in vivo studies for therapeutic as well as cosmetic effects.

**Table 1:** In vitro studies on therapeutic effects of thermal water for skin diseases

Reference	Thermal water source	Composition	Study modality	Thermal water as whole or formulation	Therapeutic effects	Results
(Borrel et al. 2019)	Uriage thermal water	sulphate, chloride, sodium, bicarbonate	*Cutibacterium Acnes*	natural mineral thermal water	preventive effects against RT4 acneic strain of C. acnes after epinephrine and norephrine exposure but limited inhibitory effects on norephine	Antimicrobial activities against catecholamines
(Gannesen et al. 2019)	Uriage thermal water	Calcium, magnesium, Sulphate, chloride, Sodium, bicarbonate	*Cutibacterium Acnes, Staphylococcus aureus*	natural mineral thermal water	increase in generation time and biomass reduction of *Cutibacterium acnes* (strain RT4 and RT5 acneic) and Staphylococcus aureus	antibiofilm activities
(Joly et al. 2014)	Uriage thermal water	Calcium, magnesium, Sulphate, chloride, Sodium, bicarbonate	Human Keratinocytes	cream	restoration of Claudin-6 expression and CAT activities after exposure to UV-B, increase in human dermal fibroblast, and reduction in lipid damage	DNA protection of cutaneous tissue to UV and antioxidant properties
(Chebassier et al. 2004)	Saint-Gervais Mont Blanc	Sodium, calcium, boron, manganese, bicarbonate, sulphate	Human Keratinocytes	natural mineral thermal water	enhance migration of keratinocytes and improvement in skin barrier functioning	wound healing effects
(Oliveira et al. 2020)	Monfortinho	Sodium, magnesium, bicarbonate, potassium, silicate	Keratinocytes and Fibroblast	natural mineral thermal water	decrease in cell metabolism and proliferation, Anti proliferative and anti-inflammatory effects	Therapeutic effect on atopic dermatitis and psoriasis
(Zoller et al. 2015)	La roche posay	Calcium, selenium, magnesium, strontium, bicarbonate	Human Keratinocytes	natural mineral thermal water	suppression of proliferation and cell damage, reverse in induction of IL-6 in human keratinocytes HaCaT cells	anti-proliferative and cell protective effects
(Rougier & Richard, 2012)	La roche posay	Calcium, selenium, magnesium, strontium, bicarbonate	Human Keratinocytes	natural mineral thermal water	decrease in lipid peroxidases production, decrease in IL-6 production, reduce production of ROS after UVB, UVA exposure	anti-inflammatory and radical scavenging activities, immunodulatory effects
(Portugal-Cohen et al. 2017)	Dead Sea	Sodium, magnesium, calcium, chloride, strontium, bromo	human-derived epidermal keratinocytes	natural mineral thermal water and anionic polysaccharide (PolluStop®)	inhibition of prostaglandin and IL-1α production	anti-inflammatory effects
(Chiarini et al. 2006a, b)	Comano thermal spa, trentino, Italy	calcium, magnesium, sulphate, bicarbonate	Human psoriatic keratinocytes	natural mineral thermal water	intracellular levels and secretions of IL-6 decrease in keratinocytes, down regulation of Cytokine (CK-16),	anti-psoriatic effects
(Chiarini et al. 2006a, b)	Comano thermal spa, trentino, Italy	calcium, magnesium, sulphate, bicarbonate	Human psoriatic keratinocytes	natural mineral thermal water	remarkable reduction expression and secretion of VEGF-A isoforms by psoriatic keratinocytes	anti-psoriatic effects
(Pra et al. 2007)	Comano thermal spa, trentino, Italy	calcium, magnesium, sulphate, bicarbonate	Human psoriatic keratinocytes	natural mineral thermal water	downregulated the intracellular levels of TNF-α, a key inducer of IL-8, IL-6, and other chemokines, abnormal differentiation of keratinocytes reduced	anti-psoriatic effects
(Grether-Beck et al. 2022)	Blue Lagoon	sodium, potassium, calcium, chloride, silica	Melanocytes	Cream composed of Blue Lagoon algae extracts	pigmented spots decreased in number, expression of α-melanocytes reduced	
(Gudmundsdottir et al. 2015; Gudmundsdottir et al. 2015)	Blue Lagoon	sodium, potassium, calcium, chloride, silica	Adult primary keratinocytes, Human monocyte-derived dendritic cells	Exopolysaccharides from *Cyanobacterium aponinum*	increase differentiation of T cells into T regulatory cells, IL-10 secretions, Reduce secretion of the chemokines CXCL10 and CCL20, Reduce keratinocytes production of LL37 inactivation of the Dectin-1 receptor, decrease recruitment of inflammatory cells, Reduce keratinocytes production of LL37 inactivation of the Dectin-1 receptor	Psoriasis treatment potential
(Aries et al. 2016)	Avene thermal spring	sodium, chloride, potassium, sulphate, silica, bicarbonate, calcium, magnesium	Normal Human Keratinocytes	thermal spring water extracted *A. dolomiae* extract	inhibited the expression of the inflammatory mediators, thymic stromal lymphopoietin, interleukin (IL)−18, IL-4R, IL-8, monocyte chemoattractant protein-3, macrophage inflammatory protein-3a, and macrophage-derived chemokine and induced the expression of involucrin, inhibited protease-activated receptor-2 activation, activated innate immunity through toll-like receptor (TLR) 2, TLR4, and TLR5 activation in human keratinocytes	anti-inflammatory, antipruritic, and immunomodulatory properties of ES0
(Eliasse et al. 2019)	Avene thermal spring	sodium, chloride, potassium, sulphate, silica, bicarbonate, calcium, magnesium	mast cells an dendritic cells	thermal spring water	reduction of the CD83, CD86, CD1a and HLA-DR molecule expression and a decrease of IL-12 and IL-23 production whereas IL-10 production was increased, reduced capacity to induce naive CD4 + T-cell proliferation and IFN-γ and IL-17 production	immunomodulatory effects
(Portales et al. 2001)	Avene thermal spring	sodium, chloride, potassium, sulphate, silica, bicarbonate, calcium, magnesium	peripheral blood mononuclear cells	thermal spring water	decrease in IL-4 production by normal peripheral blood lymphocytes, clinical features as well as the immunological Th2 profile of atopic dermatitis changes, lympho-proliferative response to some mitogens. IL-2 and IFN-γ production enhanced	anti-inflammatory and immunomodulatory effects
(Boisnic et al. 2004)	Avene thermal spring	sodium, chloride, potassium, sulphate, silica, bicarbonate, calcium, magnesium	Skin fragment from plastic surgery	thermal spring water	decrease in edema and, TNF alpha and vasoactive intestinal peptides induced dilated vessels	anti-inflammatory effects
(Castex‐Rizzi et al. 2011)	Avene thermal spring	sodium, chloride, potassium, sulphate, silica, bicarbonate, calcium, magnesium	Human endothelial Cells	thermal spring water	significant inhibition of the TNF alpha-induced E-selectin and ICAM-1 expression	anti-inflammatory effects
(Zoller et al. 2015)	Avene thermal spring	sodium, chloride, potassium, sulphate, silica, bicarbonate, calcium, magnesium	Human Keratinocytes	thermal spring water	reversed the induction of interleukin-6 in HaCaT keratinocytes	antiproliferative and anti-inflammatory properties
(Vaz et al. 2022)	protoguese natural mineral water	sulfurous/bicarbonate/sodic (SBS)	Murine skin fibroblast and macrophages	thermomineral water	increase in cell viability, superoxide dismutaseSOD activities and wound healing, anti-senescence activity	
(Gobbi et al. 2009; Mirandola et al. 2011)	Exogenous sulfur source (NaSH)	Sulfur	in vitro study immortalized human keratinocytes	Exogenous sulfur source (NaSH)	reduction in cell proliferation and suppression of IL-17, IL-8, IL-22 by downregulation of MAPK/ERK signaling pathways,	anti-inflammatory effects by reducing psoriatic lesion
Szabo et al. 2024	Szigetvár medicinal water	alkali-bicarbonates, sodium chloride and significant quantity of organics	in vitro study-HaCaT cell lines	Szigetvár medicinal water	reduce oxidative stress caused by dithranol in HaCaT cells by reducing malondialdehyde (MDA) production and suppressing cytokines IL-6, IL-8, TNF-α, GM-CSF expression	anti-oxidative effect against side effects of dithrnol

**Table 2:** In vivo clinical studies evaluating the therapeutic effects of thermal water for skin diseases

Reference	Thermal water source	Composition	Study	Thermal water as whole or formulation modeof application	Therapeutic effects	Results
(Léauté-Labreze et al. 2001)	Salies-de-Béarn	calcium, bicarbonate, magnesium	Randomized, controlled, comparative study	Patients were randomly assigned to 1 of 3 treatments: spa water alone (group A); UV-B 311-nm phototherapy alone (group B); and a combination of the 2 therapies (group C), 5 days a week for 21 days	Reduction in psoriasis area severity index (PASI)	Therapeutic effect on psoriasis
(Almeida et al. 2019)	Monfortinho	Sodium, magnesium, bicarbonate, potassium, silicate	minimized and double-blind study	Cream application on 30 Psoriasis, atopic dermatitis and eczema patients skin twice daily for 28 consecutive days	decrease in pruritic area and improvement in erythema	skin hydration effect to treat eczema and psoriasis
(Seite et al. 2013)	La roche posay	Calcium, selenium, magnesium, strontium, bicarbonate	clinical study	Ambophenol, Neurosensine, and La Roche-Posay thermal spring water formulated product tested on rosacea patients, applied twice daily for 8 weeks as monotherapy or adjunctive therapy	after exposure to sodium lauryl sulfate blood flow reduced	anti-inflammatory effects
(Faga et al. 2012)	Comano thermal spa	calcium, magnesium, sulphate, bicarbonate	In vivo experimental study (new zealand white rabbits wound model)	22 animals were enrolled and wound A,B, C were gauzes with a monolayer petrolatum, sterile saline and comano thermal water respectively. In 1 week, 24 punch biopsies performed for histological examination	fast regeneration of multilayer epithelial cells with comano thermal water gauzes, increase in migration and proliferation of keratinocytes, attenuation of regenerated collagen	skin regeneration and wound healing
(Grether-Beck et al. 2008)	Blue Lagoon	sodium, potassium, calcium, chloride, silica	In vivo experimental study on healthy human volunteers	topical treatment of healthy human skin (*n* = 20) with a galenic formulation containing all three extracts (silica mud, 2 types of algae extract	reduction of transepidermal water loss, inhibited UVA radiation-induced upregulation of matrix metalloproteinase-1 expression	improve skin barrier function and or event premature cell ageing
(Eysteinsdottir et al. 2014)	Blue Lagoon	sodium, potassium, calcium, chloride, silica	Randomized Clinical trial	68 psoriasis patients went under geothermal water bath and AND/OR NB-UVB therapy -NB-UVB treatment for 3 times a week for 6 weeks	PASI score was significantly improved in group treated with adjunctive therapy compared to monotherapy	anti-psoriatic effects
(Hirabayashi et al. 2018)	Avene thermal spring	sodium, chloride, potassium, sulphate, silica, bicarbonate, calcium, magnesium	in vivo experimental study on mice	Cream Application for 4 weeks on hind paw of mice and then Inflammation induced on mice paw by injection of complete Freund's adjuvant (CFA)	no reduction in inflammation induced by complete Freund’s adjuvant (CFA), reduction in pain (anti-nociceptive effects) was observed	no anti-nociceptive, antioxidant, analgesic effects
(Ribet et al. 2008)	Avene thermal spring	sodium, chloride, potassium, sulphate, silica, bicarbonate, calcium, magnesium	open labelled multicenter comparative study	Avene water cream or trolamine 5 times application for ten weeks on skin of patients of radiation dermatitis due to cancer therapy-dermo-cosmetic product	improved quality of life (QoL), improvement in hand eczema, eczema dermatitis symptoms improved	effective for contact dermatitis and climatic dermatitis
(Casas et al. 2011)	Avene thermal spring	sodium, chloride, potassium, sulphate, silica, bicarbonate, calcium, magnesium	clinical observational trial	18 atopic dermatitis and 39 psoriatic patients did balneotherapy for 3 weeks	inhibitory effects on S. aureus colonization, decrease in clinical symptoms 8(IL-8) expression reduced, improve PASI, SCORAD score	Anti-inflammatory properties, atopic dermatitis and psoriasis treatment
(Lee et al. 2014)	hae-undae thermal spring	/	In vivo study on psoriasis murine model	30 mice in 5 groups: Gp 1, normal control mice; Gp 2, oxazolone-induced atopic dermatitis mice sacrificed day 0; Gp 3, oxazolone-induced atopic dermatitis mice with distilled water bath for 1 week; Gp 4, oxazolone-in duced atopic dermatitis mice with mineral water balneo therapy for 1 week; Gp 5, oxazolone-induced atopic dermatitis mice without treatment sacrificed at day 7	improvement in skin erythema and scaling, Normalized T-cell proportions, reduction of mRNA levels of IL-17 A and IL-23, serum level of IL-4 and IL-5 significantly decreased	anti-inflammatory and immunomodulatory effects, psoriasis treatment
(Lee et al. 2016)	Deokgu thermal spring	sodium, Fluoride, bicarbonate	Clinical study-In vivo study on atopic dermatits hairless murine model-	nine imiquimod induced psoriasis mice divided in control group of 3 sacrificed at first day, and other 3 went under balneotherapy and other 3 bath with distilled water daily for 2 week for 5 min	increased expression of regulatory T cells, decrease lesional mRNA level of IL-33 and reduced expression in lesional IL-33 mRNA but increased cell count of CD4 + Foxp3 + regulatory T cells	immunomodulatory effects
(Bajgai et al. 2017)	Tae Chang thermal mineral water	potassium, magnesium, calcium, chlorine, bicarbonate, chloride, silica, iron, copper, fluoride, zinc	Clinical study-on atopic dermatitis induced hairless mice model-	50 mice in 5 groups: Normal control group, Negative control group treated with DNCB only + distilled water bathing, Positive control group treated with DNCB + 0.1% tacrolimus ointment + distilled water bathing, 100% pure high concentration mineral water (PHMW) group treated with DNCB + PHMW bathing, and 10% diluted high concentration mineral water (DHMW) group treated with DNCB + DHMW bathing	scratching behavior reduce, Reactive oxygen species decreased, inflammatory interleukin (IL)−1β, IL-13 and tumor necrosis factor-α were significantly inhibited, GPx activity increases, Malandialdehyde level decreased	anti-inflamatory and immunomodulatory effects
(Taieb et al. 2011)	Avene thermal spring	sodium, chloride, potassium, sulphate, silica, bicarbonate, calcium, magnesium	longitudinal observational study,	386 adult and paediatric patients of atopic dermatitis and 262 psoriasis patients went under Hydrotherapy and hydropinotherapy for 3 weeks 6 days a week	accessing points-dermatology life quality index (DLQI) and the Short-Form-12 Health Survey (SF-12) generic questionnaire were improved	psoriasis and atopic dermatitis treatment
(Giannetti 2005)	Avene thermal spring	sodium, chloride, potassium, sulphate, silica, bicarbonate, calcium, magnesium	controlled clinical trial	hydrotherapy	improvement in SCORAD index 47.8%, insomnia 66% and pruritis 41% after treatment	Atopic dermatitis improvement
(Pigatto 2005)	Avene thermal spring	sodium, chloride, potassium, sulphate, silica, bicarbonate, calcium, magnesium	In vivo study	Avene thermal water formulated emollient and spray	improvement in symptoms of atopic dermatitis (peeling, itching, pruritis and erythema)	Atopic dermatitis improvement
(Merial-Kieny et al. 2011)	Avene thermal spring	sodium, chloride, potassium, sulphate, silica, bicarbonate, calcium, magnesium	8 years observational study	(n = 5916) atopic dermatitis and (n = 4887) psoriatic patients went under hydrotherapy for 20 min for 3 weeks at 32 °C	improvement in SCORAD, pruritis, dryness and erythema, infiltration, psoriasis area and severity index	Psoriasis and Atopic dermatitis improvement
(Tarroux et al. 2002)	Avene thermal spring	sodium, chloride, potassium, sulphate, silica, bicarbonate, calcium, magnesium	clinical observation study	10 Atopic dermatitis patients underwent showers and spray baths for 20 min for 2 weeks, similarly 10 healthy controls were tested in parallel	SCORAD score improved and enzymes proteases, b-glucocerebrosidase, phospholipase A2 were similar to healthy subjects after treatment	Atopic dermatitis improvement
(Dikova et al. 2016)	La roche posay	Calcium, selenium, magnesium, strontium, bicarbonate	Observational study	100 Atopic dermatitis patients went under high pressure filiform showers, baths, facial and body spray treament and hydropinotherapy for 3 weeks,	significant improvement in itching, peeling and xerosis, dermatology life quality index (DLQI), eczema area and severity index (EASI) were also improved	Atopic dermatitis improvement
(Geat et al. 2021)	Comano thermal spa	calcium, magnesium, sulphate, bicarbonate	Observational study-	867 children suffering from atopic dermatitis underwent full body immersion balneotherapy for 2–20 min for 2 weeks at 27.7 °C	reduce the disease severity and improvement of SCORAD score in pediatric patients	Atopic dermatitis improvement
(Farina et al. 2011)	Comano thermal spa	calcium, magnesium, sulphate, bicarbonate	Open randomized Clinical trial,	104 Children with Atopic dermatitis treated with balneotherapy (n = 54) and other group treated with topical corticosteroids tacrolimus (n = 50) for 2 weeks	investigator global assessment (IGA), patients'self global assessment (PSGA), children's dermatology life quality index (CDLQI) and family dermatitis impact questionnaire (FDIQ) improvement was similar in both groups, whereas SCORAD was improved more in tacrolimus group	improvement was similar in balneotherapy and corticosteroids treated group, the relapse rate was less in balneotherapy patients, Atopic dermatitis improvement
Harari et al. 2000	Dead Sea	Sodium, magnesium, calcium, chloride, strontium, bromo	retrospective study	1718 atopic dermatitis patients- balneotherapy and climatotherapy (4 weeks)	clearance of atopic dermatitis more than 95%	
Adler-Cohen et al. 2012	Dead Sea	Sodium, magnesium, calcium, chloride, strontium, bromo	prospective study-clinical trial	49 Adult Atopic dermatitis patients underwent climatotherapy for 20 min twice a day for median of 28 days	improved SCORAD score index in AD patients, quality of life was improved analyzed by Skindex-29	
Brandwein et al. 2019	Dead Sea	Sodium, magnesium, calcium, chloride, strontium, bromo	clinical experimental study	35 Atopic dermatitis patients underwent climatotherapy for 3 weeks for microbiome analysis, 10 control run in parallel	temporal shifts of the atopic dermatitis skin microbiome improving skin microbiota reducing pathogenic microbes	effective for different skin diseases treatment
Marsakova et al. 2020	Dead Sea	Sodium, magnesium, calcium, chloride, strontium, bromo	long term follow up study	4 weeks climatotherapy, 72 children suffering from atopic dermatitis divided into 3 groups and treated climatotherapy and steroids treatment comparison in different period of time	climatotherapy and steroids treatment revealed 87.5 and 86.1% improvement of Atopic dermatitis, respectively	effective for Atopic dermatitis treatment
(Martin et al. 2015)	La roche posay	Calcium, selenium, magnesium, strontium, bicarbonate	open labelled study	54 psoriatic patients underwent 3-week selenium-rich water balneotherapy, hydropinotherapy and high-pressure filiform showers, baths, facial, and body spray treatments	significant improvement in microbiome diversity, severity of Psoriatic lesion, and disease severity, colonization of Xanthomonadaceae was increased resulting in control of psoriasis	effective for psoriasis treatment
(Beylot-Barry et al. 2022)	La Roche Posay, Uriage, Saint-Gervais, Avène, and Molitg-Les-Bains (Randomized controlled trial)	/	multicenter, open-label, randomized trial	Adult psoriasis patients,-a filiform shower (water pressure of 4 to 15 bars), followed by balneotherapy in a pool (simple or bubbling bath for 20 min), full body and facial sprays (5 to 10 min), and localized treatment (bath, spray, showers,	persistent improvement of DLQI, VQ-Dermato, and pruritus, quality of life (QoL) upgraded and psoriasis symptoms improved	
(Leaute-Labreze et al. 2001)	Salies-de-Béarn	sodium, magnesium, sulfur, chlorine, potassium, iron, phosphorous, manganese	Randomized, controlled, comparative study,	spa water alone (group A n = 22); UV-B 311-nm phototherapy alone (group B n = 21); and a combination of the 2 therapies (group C n = 24), intervention duration 5 days a week for 21 days	significant reduction of (PASI) was reported in combined treatment of balneotherapy and phototherapy	balneotherapy and phototherapy combined treatment significantly effective for psoriasis management
(Borroni et al. 2013)	levico and vetriol spa	arsenical-ferruginious water	double-blind, randomized, placebo-contralaterally-controlled trial	34 psoriasis patients water wet packing daily for 20 min for 12 consecutive days	histopathological and immuno-histochemical parameters improved with reduction in psoriatic lesions	effective for psoriasis treatment
(Pagliarello et al. 2012)	Comano thermal spa	calcium, magnesium, sulphate, bicarbonate	Observational study	full body bath for 20 min 6 days a week for 2 weeks along with UV/B phototherapy upto 0.05–0.1 J/cm2 to psoriatic patients	improvement in SkinIndex and SPASI score	balneotherapy and phototherapy combined treatment significantly effective for psoriasis management
(Tabolli et al. 2009)	Comano thermal spa	calcium, magnesium, sulphate, bicarbonate	Observational prospective study	111 patients enrolled, in first group (n = 66) psoriasis patient treated for 2 weeks of balneotherapy with phototherapy and in second group (n = 45) only 2 weeks of balneotherapy	social functioning, mental health, SPASI score were significantly improved, QoL improved	effective for psoriasis treatment
(Peroni et al. 2008)	Comano thermal spa	calcium, magnesium, sulphate, bicarbonate	prospective, non-randomized, open study	300 patients enrolled, in first group (n = 40, n = 84) psoriasis patient treated for 1 to 2 weeks of balneotherapy with phototherapy and in second group (n = 77, n = 40) only 1 to 2 weeks of balneotherapy, one to two bath for 20 min at 37 °C for 2 weeks	comparatively more significant reduction of (PASI) was reported in balneophototherapy group	effective for psoriasis treatment
(Eysteinsdottir et al. 2014)	Blue Lagoon	sodium, potassium, calcium, chloride, silica	Randomized trial	68 patients enrolled-balneophototherapy 3 times a week for 6 weeks, balneophototherapy daily for 6 weeks, phototherapy for 6 weeks	quality of life and histopathological parameters improved, PASI 75 and PASI 90 in first treatment group was more pronounced,	balneophototherapy more effective for psoriasis treatment
(Bodemer et al. 2011)	Avene thermal spring	sodium, chloride, potassium, sulphate, silica, bicarbonate, calcium, magnesium	prospective, open‐label, multi-centre study	20 children and 24 adult patients enrolled-hydrotherapy of ichthyosis patients for 3 weeks and follow up visit 3 and 6 months later at the reference	improved Dermatology life quality index (DLQI) score and ichthyosis severity reduced even after 3 and 6 months of treatment disease was controlled	Ichthyosis treatment
(Boisnic et al. 2004)	Avene thermal spring	sodium, chloride, potassium, sulphate, silica, bicarbonate, calcium, magnesium	In vivo study	oral bath, vaporization, gingival showers, compresses and hydropinotherapy	symptoms of ichthyosis (itching, erosion, erythema)	improved, patients do not need to take any analgesic, corticosteroids, Ichthyosis treatment
Gebretsadik 2023	Ethopian Hot springs		A single arm prospective cohort study	1320 study participants visiting hot-spring and staying atleast 3 days were enrolled-Balneotherapy of enrolled participants with skin lesions, psoriasis, and eczema with minimum of 4 days and extended upto 30 days treatment	Improvement in PASI score 4.4 to 0.4, 73.3% patients completely relieved, 18.3% partially improved, 8.5% no improvement	Psoriasis, eczema, skin lesions treatment
(Portugal-Cohen et al. 2011)	Dead Sea	Sodium, magnesium, calcium, chloride, strontium, bromo	double-blind controlled study	in total 86 children enrolled-3 experimental groups: Dead sea water and Dead sea Mud and other components (DM) and Dead sea mud and water with other components (TP) and Control emollient (E) without dead sea component, Twice a day application of formulation according to relevant group, treatment duration 6 week	TP was the most effective regarding transepidermal water loss (TEWL), stratum corneum hydration (SCH) and Objective Severity Assessment of Atopic Dermatitis (OSAAD) compared to DM and E, Only TP improved TEWL and SCH	skin barrier function, AD skin treatment

**Table 3:** In vitro studies on the cosmetic effects of thermal water for skin

Reference	Thermal water source	Composition	Cell lines	Thermal water/formulation	Therapeutic effects	Results
(Gueniche et al. 2022a)	vichy thermal water	Magnesium, calcium, potassium, sodium, sulphate	human epidermal keratinocyte	natural mineral thermal water	increase in expression of β-defensin-4 A and S100 A7, downregulation of interleukins IL-8, IL-23P40, IL-12, Increase in IL-10, IL10/IL12, increase in filggarin, transglutaminsase	immune defence and skin barrier, protective effects on Langerhen cells against UV radiation, antimicrobial peptide defence
(Tacheau et al. 2018)	vichy thermal water	Magnesium, calcium, potassium, sodium, sulphate	human keratinocytes	natural mineral thermal water	increase in cutaneous homeostasis genes expression,	skin hydration, DNA repair, antioxidant defense, differentiation in cell proliferation
(Joly et al. 2014)	Uriage thermal water	Calcium, magnesium, Sulphate, chloride, Sodium, bicarbonate	Normal human skin explants	natural mineral thermal water	epidermal expression of aquaporin-3, claudin-6 expression, filaggrin and claudin-4 enhanced,	dry skin hydration
(Verdy et al. 2012)	Uriage thermal water	Calcium, magnesium, Sulphate, chloride, Sodium, bicarbonate	human keratinocytes	natural mineral thermal water	effect on vitamin C transporter 1 expression and taurine transporters	skin Défense action against different stressors such as ultravoilet-B (UV-B) irradiation, aging, dehydration
(Nunes et al. 2019)	Cro	potassium, sodium, silica, calcium, magnesium, bicarbonate	human keratinocytes	gel	skin improvement (decrease in roughness, scaling), smoothness increases, decrease in TEWL, increase in hydration and human dermal fibroblasts adhesion	cell hydration and proliferative effects
(Nicoletti et al. 2019)	Comano thermal spa, trentino, Italy	calcium, magnesium, sulphate, bicarbonate	Human Skin biopsies culture	natural mineral thermal water	Collagen and elastic fiber regeneration, cell proliferation,	skin regeneration
(Grether‐Beck et al. 2008)	Blue Lagoon	sodium, potassium, calcium, chloride, silica	Primary human epidermal keratinocytes	cream made of algae extract and silica mud	induction of involucrin, loricrin, transglutaminase-1 and filaggrin gene expression in primary human epidermal keratinocytes, increase in collagen 1 A1 and 1 A2 gene expression	improve skin barrier function and prevent premature skin aging
Pinto-Ribeiro et al. 2024	Chaves Thermal water	Bicarbonates, carbonates, carbon dioxide rich	human epidermal keratinocyte and Human primary dermal Fibroblasts	Thermal water application	PM expxosed HaCaT cells treated with Chaves thermal water and IL-6 levels reduce tenfold compared to control, inhibition of elastase in nHDFs cells	Anti-inflammatory, anti-aging properties
(Portugal-Cohen et al. 2011)	Dead Sea	Sodium, magnesium, calcium, chloride, strontium, bromide	Human skin fragment	cream	hydration of stratum corneum, OSAASD and TEWL score increased	antioxidants and cell viability increased, uric acid content also increased, therapeutic ability to treat Atopic dermatitis
(Nicoletti et al. 2019)	Comano thermal spa	calcium, magnesium, sulphate, bicarbonate	Human fibroblasts	natural mineral thermal water	anti-inflammatory effects by reducing dermal cell infiltration	wound healing and tissue regeneration potential
Mourelle et al. 2024	La Solia thermal spring water	chloride, sulfate, sodium, zinc, copper, barium, bicarbonate, sodium, calcium	human epidermal keratinocyte HaCaT	thermal spring water	thermal water attenuated the levels of pro-inflammatory mediators (IL-6, IL-1, TNFα, NF-κB, and CCL1), at the level of gene expression	skin anti-irritant potential

**Table 4:** In vivo clinical studies evaluating the cosmetic effects of thermal water for skin

Reference	Thermal water source	Composition	Study	Thermal water as whole or formulation	Therapeutic effects	Results
(Gueniche et al. 2022a, b)	vichy thermal water	80% vichy mineral water, 5% V. filiformis (VfeV), 4% niacinamide (vitamin B3), 0.4% hyaluronic acid, and 0.2% vitamin E	Clinical trial	50 woman applied Minéral 89 Probiotic Fractions (M89PF) cream for 12 weeks	Upregulation of skin antioxidant defense system, lightening skin pigmentation effect, skin barrier effects	improvement in recovery of microbiome after exposure to acute stress conditions by using harsh cleansers, skin depigmentation effect
(Ferreira et al. 2010)	São Pedro do Sul	chloride, fluoride, bicarbonate, sulfate, sodium, silica	Clinical trial 17 healthy Caucasian volunteers	skin of 17 volunteers irritated with sodium lauryl sulfate and trated with São Pedro do Sul (SPS) thermal water and kept under occlusion for 48 h for observation	reduction in Transepidermal water loss (TEWL)	anti-irritant effect on skin
(Goldman et al. 2007)	Avene thermal spring	sodium, chloride, potassium, sulphate, silica, bicarbonate, calcium, magnesium	double blind monocentric comparative study	patients given photodynamic therapy were treated with thermal water spray and other group with comparative water as control for 6 days	improvement in pain, erythema, pruritis	anti-irritant and soothing effects
(Grether-Beck et al. 2022)	Blue Lagoon	sodium, potassium, calcium, chloride, silica	monocentric, double blind vehicle controlled split face study	60 volunteers with pre-existing facial pigment spots used Cream composed of Blue Lagoon algae extracts twice daily for 1 week	pigmented spots decreased in number, expression of α-melanocytes reduced	
(Mias et al. 2020)	Avene thermal spring	sodium, chloride, potassium, sulphate, silica, bicarbonate, calcium, magnesium	Ex vivo and clinical study	40 participants divided in 2 groups-thermal spring water and mineral rich spring water group, water Spray on peeling skin of selected participants	improvement in skin redness, peeling and sensitivity, skin hydration	skin hydration and anti-irritant effect
(Benedetti et al. 2009)	saturnia spring	sulfates, calcium, magnesium, sodium, pottasium, flouride, chloride, bicarbonates, iron	In vivo study-	healthy humans (hydropinotherapy of sulfurous thermal water	Increase in antioxidants capacity, reduced levels of protein and lipids oxidation and advance oxidation Protein products (AOPP)	antioxidative capacity
Pinto-Ribeiro et al. 2024	Chaves Thermal Water	Bicarbonates, sodium, carbon dioxide rich	In vivo/in vitro (in vivo microbiota study of enrolled healthy humans)	Application on arm skin of 23 volunteers	Significant skin hydration, reduced TWEL, health skin microbiota	Cosmetic effects
(Altaany et al. 2019)	Al- Hammah sulfurous springs	calcium, chloride, phosphate, nitrate, ammonia, magnesium, sodium, potassium, sulfur, bicarbonate,	Clinical trial	residents in vicinity of sulfurous thermal water blood samples for total oxidative stress (TOS) and Oxidative stress index (OSI) analysis	total antioxidant capacity (TAC), oxidative stress index (OSI) and total oxidative stress (TOS). The results demonstrated significantly higher TAC and lower (OSI) and (TOS)	antioxidative capacity
(Alirezai et al. 2000)	Avene thermal spring water	sodium, chloride, potassium, sulphate, silica, bicarbonate, calcium, magnesium	Multicenter, open labeled comparative study, Clinical trial	Group 1: Thermal water + retinoic acid, Group 2: Retinoic acid only	Desquamation of acne, thermal water improved the tolerance of retinoic acid caused itching	Acne spots desquamation
(Kulisch et al. 2023)	Lake Heviz	sulphurous thermal water	open label pilot study	thermal water bath 5 days a week for 3 weeks	significant improvement in PASI score, increase colonization of Leptolyngbya genus and decrease in Flavobacterium genus	healthy skin microbiota increase, psoriasis treatment effectiveness
(Deleuran et al. 2020)	Avene thermal spring	sodium, chloride, potassium, sulphate, silica, bicarbonate, calcium, magnesium	open labeled real world study	*Aquaphilus dolomiae *extract containing emollient cream applied on 5910 xerosis/pruritus patients skin two times a day for 7 days	improvement in SCORD, DLQI, Sleep quality, reduction in pruritis and xerosis, remarkable improvement in itching duration	soothing properties, anti-irritant effects
(Vendrely et al. 2022)	Avene thermal spring	sodium, chloride, potassium, sulphate, silica, bicarbonate, calcium, magnesium	open labeled real world study	319 xerotic cancer patients used *Aquaphilus dolomiae* extract containing emollient cream for 4 week,	Xerosis severity reduced, DLQI score improvement, well tolerated emollient even in anticancer therapy patients	skin hydration, anti-irritant, xerosis and pruritis treatment
(Barolet et al. 2009)	Avene thermal spring	sodium, chloride, potassium, sulphate, silica, bicarbonate, calcium, magnesium	split face comparative Clinical study	thermal water spray effect on fractional resurfacing laser skin 6 times a day for 2 days	psoriatic pain management, remarkable reduction in pruritis and erythema	anti-irritant and soothing effects

## Results and discussion

### *Thermal water and skin disease treatment-*in vitro* approach*

**Fig. 1:**
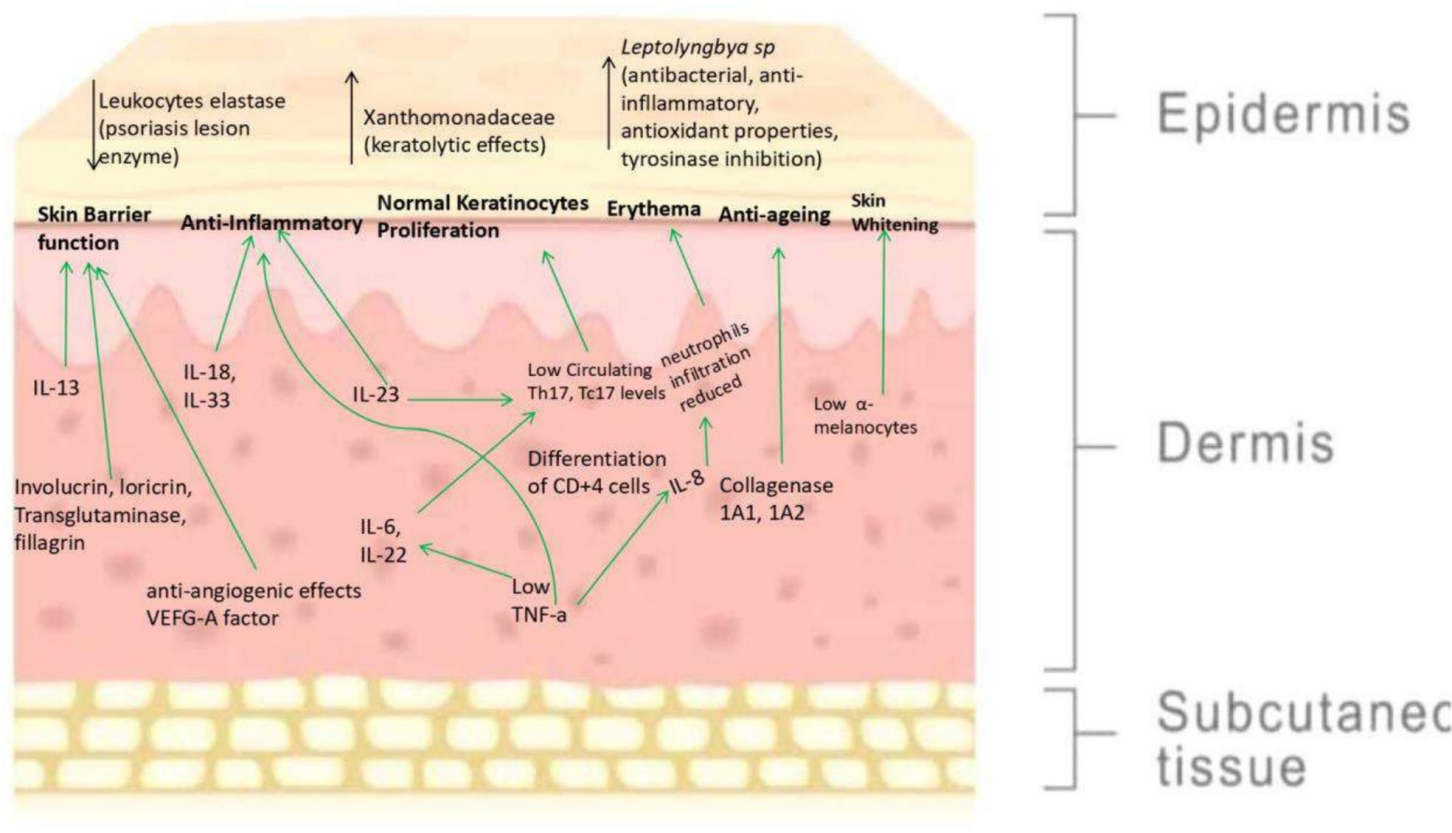
Summary of the biochemical effects of thermal water treatment on skin based on a literature review (Martin et al. 2015; Xiao et al. 2021; Pra et al. 2007; Chiarini et al. 2006a; Mirandola et al. 2011; Gobbi et al. 2009; Aries et al. 2016; Eliasse et al. 2019; Portugal-Cohen et al. 2017)

Most in vitro studies have investigated the effects of thermal water on human keratinocytes and foreskin fibroblasts. Most of the in vitro studies reported thus far have focused mainly on immune-mediated inflammatory skin disease psoriasis (Table 1). Psoriasis is a skin condition in which intralesional T lymphocytes increase the production of keratinocytes, enhancing disease effects. Moreover, during disease pathogenesis, Th1/Th17 lymphocytes trigger the production of the interleukins IL-12 and IL-17, leading to keratinocyte-mediated IL-8 production (Fig. 1) (Marzano et al. 2018). Considering that psoriasis is dependent on an angiogenic mechanism, A. Chiarini et al. (2006a, b) studied the release of the vascular endothelial growth factor VEGF-A and its expression using Comano thermal water rich in bicarbonate, calcium, and sodium. They reported the effects of thermal water on the expression and secretion of VEGF-A; in addition, they reported a significant reduction in IL-6 and cytokeratin-16 in another similar study (Chiarini et al. 2006a). In the subsequent study with same type of thermal water (Comano thermal water) demonstrated a significant reduction in the levels of IL-8, proinflammatory cytokines and TNF-α in psoriatic keratinocytes (Pra et al. 2007). Lee et al. (2012) investigated the effects of Yong-gung oncheon thermal water (rich in calcium, sulfur, selenium, and magnesium) on articulated symptoms of inflammatory skin diseases via human keratinocytes HaCaT cell lines. They reported low differentiation of CD4 + T cells and significant inhibition of the most pronounced inflammatory interleukins, IL-6 and IL-8, after 1, 4, 10, and 24 h of treatment with thermal water. In another study, on turkey thermal spa water, anti-inflammatory and angiogenic effects were observed in the human keratinocytes cell line HaCaT, which is used to treat psoriasis and rosacea. HaCaT cells were incubated with 2 types of thermal water at a 10% concentration for 72 h. The results confirmed the suppression of tumor necrosis factor (TNF-α) and IL-1α gene expression and of VEGF.

Sulfur is known to be effective for the treatment of psoriasis and can penetrate the skin barrier. Therefore, a sulfur-rich thermal water exogenous source (NaSH) was studied by Mirandola et al. (2011) and Gobbi et al. (2009) using immortalized human keratinocyte cultures. They reported a reduction in cell proliferation and the suppression of IL-17, IL-8, and IL-22 through the downregulation of MAPK/ERK signaling pathways and anti-inflammatory effects by minimizing psoriatic lesions.

Moreover, the effects of solar or artificial UV radiation on the skin were studied against Gyoparos and Hungarian Kakasszék spa thermal water as a therapeutic agent. UV-irradiated HaCaT cells were incubated with different organic extracts of both types of thermal water. Gyoparos water prevents UV radiation-induced DNA lesions, providing protection against UV radiation (Gerencser et al. 2019).

The use of thermal water has been well recognized for its therapeutic effects on skin disease (Oliveira et al. 2020). The monfortinho thermal water is hyposaline with high concentrations of calcium, magnesium, sodium, silica, and bicarbonate ions. Sodium and silica represent > 50% of the total mineralization of monfortinho water. Owing to the enrichment of sodium and silica, it has beneficial properties for skin disease treatment.

Oliveira et al. (2020) studied the effects of monthly exposure to thermal water on skin homeostasis in keratinocytes, fibroblasts, and macrophage lines. They reported a significant reduction in the cellular metabolism of macrophages (25%), keratinocytes (60%), and fibroblasts (45%) in response to treatment with thermal water. Cell proliferation was also reduced in all the cell lines exposed to thermal water. Therefore, these findings suggest a remarkable therapeutic potential for treating psoriasis and atopic dermatitis. The overall biochemical pathway of the therapeutic effects of thermal water on the skin is summarized in Fig. 1.

## Thermal water and skin disease in vivo approach

The skin diseases most commonly treated with thermal water include atopic dermatitis, psoriasis, ichthyosis, lichen planus, and acne vulgaris but with prime focus on psoriasis and atopic dermatitis (Table 2). Recently, Protano et al. (2024) and Moini Jazani et al. (2023) reviewed the efficacy of balneotherapy effects on atopic dermatitis and psoriasis with narrow selection criterion targeting only in vivo studies and specifically using balneotherapy approach. But the other diseases such as acne vulgaris, ichthyosis, lichen planus were not studied. Moreover, the cosmetic effects were out of scope of these review. Therefore, this comprehensive review provides a deep insight into therapeutic effects of thermal water for management of different skin diseases and also evaluates cosmetic properties of thermal water using in vivo as well as in vitro studies. The treatment efficacy depends on many factors, such as treatment modality, disease severity, ancillary disease conditions, thermal water composition, and treatment duration. In the studied period, the thermal spa waters most studied for skin diseases include La Roche-Posay, France; Vichy, California; Avène, France; Vetriolo and Levico, Italy; Cro, Portugal; Monfortinho, Portugal; Salies de Bearn Spa, France; Uriage, France; Comano, Italy; the Dead Sea, Israel; and the Blue Lagoon, Iceland.

### Thermal water in atopic dermatitis management

Atopic dermatitis (AD) is an anti-inflammatory disease of the skin that impairs quality of life, with lesions on visible parts of the skin and poor sleep quality (Bender et al. 2008) (D. Geat et al. 2021). The factors responsible for AD pathogenesis include both environmental and genetic factors. An emerging treatment with thermal water rich in minerals has been recognized for its soothing and healing properties on the skin. The studies exploring thermal water as a treatment for AD highlight its potential benefits, including reducing inflammation and improving life quality with minimal side effects. The holistic approach utilizes the unique mineral composition of water from thermal springs known for their therapeutic properties.

**Fig. 2:**
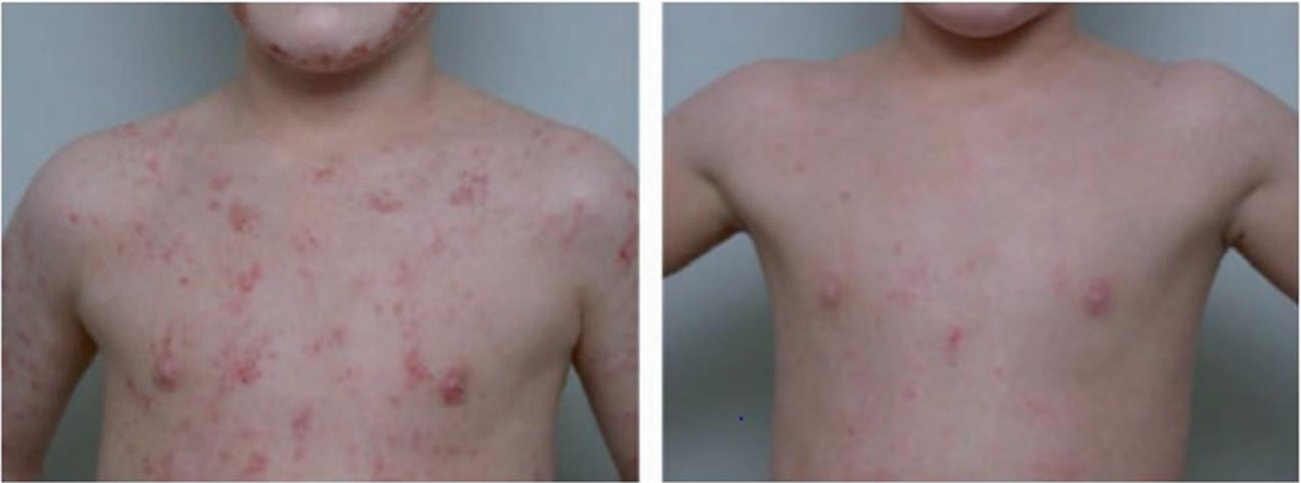
Before and after 3 weeks of Avène thermal water hydrotherapy-based improvement in atopic dermatitis (Merial‐Kieny et al. 2011). Reproduced with permission from Merial-Kieny et al. 2011. © Wiley. License Number: (6010820094848)

In a study conducted by Taieb et al. (2011), the largest cohort of dermatitis patients was treated with Avène thermal water. The duration of hydrotherapy was 3 weeks, and patients reported significant improvements in quality of life and sleep. Overall, a 41.6% improvement in the severity of AD and pruritis was observed. A similar observational study on a large cohort of AD patients reported remarkable improvements in the SCORD index (47.8%), insomnia (66%), and pruritis (41%) after treatment (Giannetti 2005). Merial-Kieny et al. (2011) reported analogous results of improvement in pruritis, the SCORAD index, and dryness in another study (Fig. 2). Treatment with Avène thermal water is also known to have inhibitory effects on IL-8 and reduce the colonization of *Staphylococcus aureus,* resulting in a reduced SCORAD index (C. Casas et al. 2011).

Pigatto (2005) studied the effects of Avène thermal water formulated as an emollient and spray on AD in 76 children and reported that the combination of an emollient and a thermal water spray effectively improved the subjective symptoms of AD, such as peeling, itching, pruritis, and erythema.

To better understand the mechanism of action of Avène thermal water in the treatment of AD, various pharmacological interventions have reported its anti-inflammatory effects, pathogenic skin microbiome alterations to nonpathogens, keratinocyte differentiation, and cytokine immunomodulatory effects (Guerrero & Garrigue 2017; Merial-Kieny et al. 2011). The enzymes proteases b-glucocerebrosidase, and phospholipase A2 play major roles in the epidermis barrier in AD pathogenesis (Redoules et al. 1999), and in AD patients treated for 3 weeks with Avène thermal water, the activities of these enzymes are similar to those of healthy subjects (Tarroux et al. 2002).

Despite the evident therapeutic properties of Avène thermal water for the treatment of AD, the exact mechanism of action is not fully understood. As Avène thermal water profoundly reduces the colonization of pathogenic bacteria such as *Staphylococcus aureus* in AD lesions, the inhibition of inflammatory interleukins (IL-8) (responsible for AD aggression and the regulation of the enzymatic activities of proteases, b-glucocerebrosidases, and phospholipase A2 to restore the AD rupture skin barrier and ameliorate AD symptoms, resulting in soothing and antipruritic effects) could be responsible for its therapeutic effects. Considering the therapeutic potential of Avene thermal water, its exact mechanism of action for treating AD should be elucidated in future research.

La Roche Posay thermal water was also reported to have significant therapeutic effects on atopic dermatitis by improving itching, peeling, and xerosis. The dermatology life quality index (DLQI), eczema area, and severity index (EASI) also improved (Dikova et al. 2016). The proposed mechanism of action of LRP-TW could involve high selenium levels in this water, as low selenium levels are known to be associated with the pathophysiology of AD (Kouhkan et al. 2006). The therapeutic properties of selenium are associated primarily with selenoproteins, which have hermetic advantages at low doses and toxic effects at high concentrations. Selenium-containing compounds ameliorate the antioxidant activities of glutathione peroxidase (GPx) and thioredoxin reductase (TrxRs), which are major players in the pathologies of different skin diseases (Roman et al. 2014). The valuable properties of selenium are studied in the context of diseases other than skin diseases; hence, future studies should further infer the underlying curative factors for its curative properties in different skin diseases, including AD.

Comano-thermal spring water is oligometallic and rich in bicarbonate, calcium, and magnesium ions (Figueiredo et al. 2023). Comano spring thermal water is famous for its therapeutic activities for different diseases. Davide Geat et al. (2021) investigated the effects of Comano spring TW on pediatric AD patients and reported significant improvement in AD severity in children with mild to severe AD conditions. Similarly, another study reported improvement in disease severity in pediatric AD patients treated with Comano TW, and the results were comparable to those in corticosteroid-treated patients (Farina et al. 2011). Despite the notable therapeutic efficacy of Comano thermal water against AD, the mechanism of action of Comano thermal water is unknown. The proposed mode of action of Comano thermal water is attributed to its anti-inflammatory properties, which restrict the production and secretion of cytokines in keratinocyte cells. This hypothesis has been extensively validated by many in vitro studies in which psoriatic keratinocytes treated with thermal water presented alterations in cytokine 16, tumor necrosis factor (TNF)-alpha expression, vascular endothelial growth factor-A expression and secretion (Chiarini et al. 2006a), and interleukin (IL)−6 and IL-8 production and secretion (Chiarini et al. 2006a)). Further studies are needed to better understand the mechanism of action of Comano thermal water for the management of disease pathogenesis.

The Dead Sea is the most pronounced thermal water source because of its wide range of therapeutic applications, which are mainly attributed to its low radiation, high UVB: UVA ratio, and low elevation (Huang et al. 2018). These geographical properties of the Dead Sea make it valuable. Moreover, the high salt and mineral concentrations (bromine, calcium, sulfide, potassium, and magnesium) resulting in the layering of salts on the water surface further enhance the therapeutic properties of Dead Sea water (Huang et al. 2018).

Furthermore, the Dead Sea is known to have therapeutic potential for treating AD. Numerous studies have reported the climatotherapy effects of the Dead Sea for the treatment of AD by improving the SCORAD score (Skindex-29) in AD patients (Adler-Cohen et al. 2012; Harari et al. 2000). A retrospective study of 1718 AD patients reported the therapeutic effects of Dead Sea climatotherapy on the clearance of AD after 4 weeks of treatment (Harari et al. 2000). In another study, climatotherapy was shown to be a suitable treatment for AD by improving the SCORAD score Skindex-29 in AD patients (Adler-Cohen et al. 2012). Moreover, Brandwein et al. (2019) reported the effects of climatotherapy on the alteration of the skin microbiome of AD patients to promote the beneficial effects of Dead Sea climatotherapy on different skin conditions, including AD. Following dead sea climatotherapy, the colonization regimens of *S. epidermidis,M. luteus* and *S. mitis* was significantly affected. The relative abundance of *S. mitis* increased, whereas the relative abundances of *S. epidermidis and M. luteus* decreased after climatotherapy in the Dead Sea*. S. mitis* and *M. luteus* are highly prevalent in the skin microbiota; nonetheless, the role of these species in skin homeostasis and health is not yet clear. In addition, the epidermis of the other bacterial species *S.* is also an important member of the healthy skin microbiota and is known to be involved in skin health, as it is known to resist colonization by *S. aureus* and immune response modulation. The *S. epidermis* has been found in mild AD lesions and nonlesional skin, suggesting that treatment with dead sea climatotherapy clears the skin by diminishing lesional and nonlesional colonization of the *S. epidermis.* Hence, climatotherapy at the Dead Sea offers a temporal shift in the AD microbiome and evidence of climatotherapy success on AD skin.

In another clinical trial, AD patients were treated with steroids and climatotherapy in the Dead Sea in 2 groups. A comparative study of climatotherapy and steroid treatment revealed 87.5% and 86.1% improvement in disease conditions, respectively (Marsakova et al. 2020).

### Thermal water in psoriasis management

Psoriasis is a chronic inflammatory skin disease with a high chance of recurrence after treatment. The prevalence of psoriasis is 0.5–4.6% in different regions of the world. Psoriasis is accompanied by physical, emotional and social distress, resulting in a low quality of life (Boros et al. 2013; Qiu et al. 2014). Thermal water treatment can be proposed as the most promising and safe treatment for psoriasis (Boros et al. 2013; Seite et al. 2013). The most studied thermal spring waters for the treatment of psoriasis include Comano, Dead Sea, La Roche Posay, Avene, Levico, vitriol, Salied de Bearn, Lake Herviz, and Blue Lagoon thermal water.

**Fig. 3:**
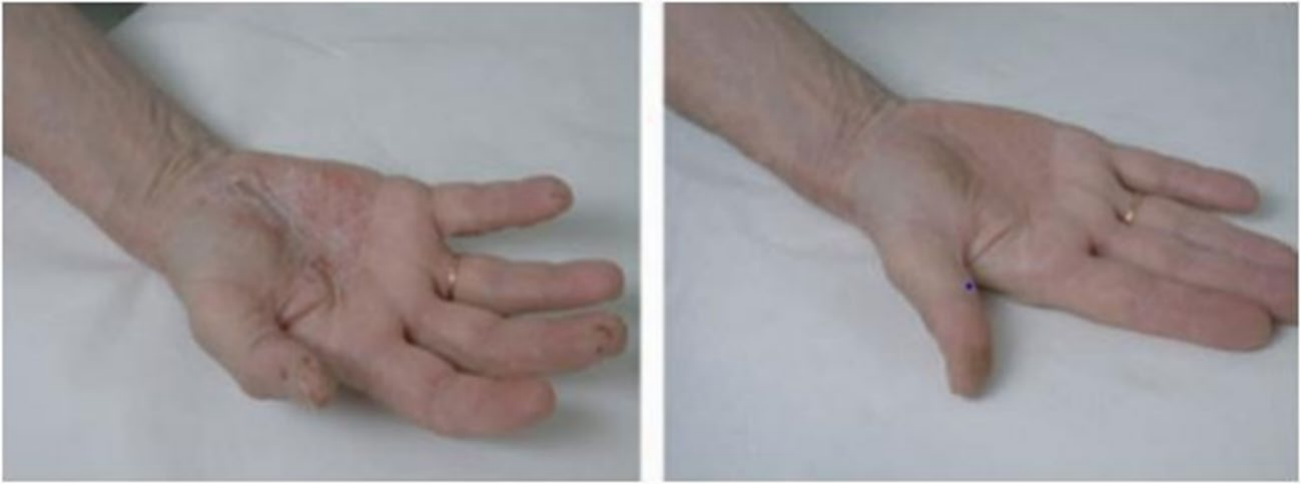
Before and after 3 weeks of Avene thermal water hydrotherapy-based improvement of psoriasis (Merial‐Kieny et al. 2011) Reproduced with permission from Merial-Kieny et al. 2011. © Wiley. License Number: (6010820094848)

Avène thermal water is well known for its antipsoriatic effects, as validated by various long- and short-term studies. A long cohort (8 years) observational study investigated the effectiveness of Avène thermal water for the treatment of psoriasis patients. The treatment comprises Avène thermal water baths at 32 °C for a total of 20 min for 6 days a week for 3 consecutive weeks with the application of an emollient with sedative and anti-inflammatory effects, spraying Avène water to remove scales, and a drinking cure. The results revealed significant improvements in erythema, infiltration, the psoriasis area, and the severity index in psoriasis patients (Merial‐Kieny et al. 2011) (Fig. 3). In another study, 3 weeks of Avene thermal water treatment improved the Dermatology Life Quality Index (DLQI) score of psoriasis patients even after 6 months of treatment (Taieb et al. 2011). Moreover, a molecular-scale study elucidated the anti-inflammatory potential of Avene thermal water through the suppression of inflammatory interleukins and reduction *in Staphylococcus aureus* colonization in psoriatic skin. The improvement biomarker in this study was the psoriasis severity index (PASI) score (C Casas et al. 2011). Similarly, Thouvenin et al. (2023) conducted a clinical trial on 26 psoriatic patients with chronic pruritus who were receiving thermal water hydrotherapy for 3 weeks and 18 psoriasis patients as controls who were receiving regular treatment with no hydrotherapy. The study reported that, compared with those in the control group, remarkable improvements in pruritus and psoriasis PASI scores were achieved by 40.4% in the treatment group. The molecular study of gene and protein biomarkers also revealed decreases in psoriasis biomarkers (PI3, S100 A7, and IL-17), inflammation biomarkers (IL-8, IL-1α, and IL-1RA), and pruritus biomarkers (TRPV1, IL-31 and CGRP1).

In summary, the results from different studies strongly demonstrate subjective and objective improvements in psoriasis and related quality of life (QoL) after treatment with Avene thermal water with a minimum of 3 weeks of balneotherapy with no relapse of disease after at least 6 months of treatment. Nevertheless, the underlying mechanism for the significant therapeutic efficacy of Avene thermal water has not yet been elucidated. Therefore, the mechanism of action of thermal water should be elucidated to determine the benefits of this medicinal water in the treatment of various skin diseases.

La Roche Posy Spring thermal water is popular for psoriasis treatment because of its rich mineral components. The clinical study evaluated the effects of the LRPT-TW filiform shower (15 bars for 3 min) and revealed that patients’ conditions improved with a reduction in psoriatic lesions. Another study (LRPT-TW) reported significant improvements in microbiome diversity, the severity of psoriatic lesions, and disease severity. The colonization of *Xanthomonadaceae* increased, resulting in keratolytic effects; therefore, the increase in colonization and diversity of *Xanthomonadaceae* could be responsible for curing psoriasis (Martin et al. 2015).

The combined intervention of filiform shower, thermal bath, facial and body spray, and localized treatment with La Roche Posay and 4 other types of thermal water (depending on the choice of the patient to join any spa center) was studied with 128 patients over 1 year. The assessment parameters used to evaluate the effectiveness of thermal water were the Perceived Stress Scale (PSS) stress score, visual analog scale (VAS) score, Dermatology Life Quality Index (DLQI), psoriasis severity index. The results revealed significant improvements in quality of life (QoL) and other parameters (Beylot-Barry et al. 2022). Hence, the antipsoriatic potential of La Roche Posay water is well known from different randomized controlled trials, but its mechanism of action is unclear and needs to be explored further.

Salies de Bearn thermal water (“salted water”) is rich in sodium and magnesium and has many therapeutic activities. It has been the least studied for dermatological applications to treat various skin diseases. To the best of our knowledge, there is only one randomized controlled trial on psoriasis treatment involving the use of salted water from salies de bearn. This research investigated the potential of salies de Bearn water to treat psoriasis with and without a UV-B phototherapy regimen. In group A, only thermal water treatment was used; in group B, only phototherapy was used; and in group C, combined balneophototherapy was tested for 21 days, 5 days a week. Treatment efficacy was assessed by the psoriasis area severity index (PASI), and a significant reduction in the PASI was reported after treatment with thermal water, and no significantly enhanced effects were observed when phototherapy was used (Léauté-Labreze et al. 2001). The treatment efficacies in groups A, B, and C were 29%, 64%, and 55%, respectively. The only study is a randomized clinical trial with many limitations and biases because 3 patients suddenly experienced adverse effects, and 1 patient experienced pulmonary infection and left the clinical trial. Furthermore, the minor improvement in group A with balneotherapy is biased, indicating that the improvement might be due to stress reduction induced by emollients and natural therapy relief. Owing to minor improvement after balneotherapy and high bias, it is not advised as a complementary treatment approach for psoriasis. Therefore, in vitro and in vivo studies should be designed to investigate the therapeutic efficacy of saline water for treating skin diseases with an explanation of its mode of action.

The thermal water of levico- and vetriol is arsenical ferruginous rich in iron and sulfates and has residues of magnesium and arsenic with a pH of 1.6. Acidic water with residues of arsenic is known to have antiproliferative effects on psoriasis keratinocytes. Furthermore, keratinocyte apoptosis has also been reported to occur in response to high concentrations of arsenic through FAS ligand pathways (Liao et al. 2009). On the other hand, the effects of iron on psoriatic patients are controversial. Some authors reported positive effects of iron on psoriasis patients, whereas others reported the opposite. Therefore, more studies are needed to explore its effects. The therapeutic effects of the arsenical-ferruginious water of Levico and Vetriol on psoriasis patients were studied. The intervention included 34 adult patients with mild to moderate psoriasis treated with Arsenical ferruginous water wet packing daily for 20 min for 12 days. The results revealed improvements in psoriatic lesions, as assessed by histopathological (hyperkeratosis score, granular layer, mitoses, papillomitoses, and dilation of blood vessels) and immunohistochemical (proliferation antigen-Ki67) parameters, in the treated group compared with the control group (G. Borroni et al. 2013).

Pagliarello et al. (2012) studied the effects of Comano thermal water on psoriasis patients with and without phototherapy treatment at a mean age of 18 years who were treated for 6 days a week for 2 consecutive weeks. The treatment modality was a full-body bath in thermal water for 20 min at a constant temperature of 27.7 °C and a pH of 7.2. In addition to the thermal water bath, patients were also given phototherapy treatment with a starting range of 0.3–0.4 J/cm^2^, with an increase of 0.05–0.1 J/cm^2^. The results revealed improvements in the SkinIndex and SPASI score after treatment.

Similarly, Tabolli et al. (2009) reported the beneficial effects of a Comano thermal bath with phototherapy to treat moderate to severe psoriasis. In this observational prospective study, 111 patients with psoriasis were treated with a thermal water bath and phototherapy (UV-B therapy) alone or in combination for 2 weeks. The assessment points included the general health questionnaire (GHQ-12), quality of life questionnaire (QoL), and self-administered psoriasis area severity index (SPASI). The social functioning, mental health, and SPASI scores were significantly improved, suggesting the beneficial effects of thermal water and phototherapy for the treatment of psoriasis patients.

Kulisch et al. (2023) studied the effects of Heviz thermal water on psoriatic skin and the skin microbiome of psoriatic and nonpsoriatic skin before and after treatment. Patients with plaque psoriasis aged 18–70 years were enrolled for 3 weeks of balneotherapy treatment at Heviz sulfurous thermal spa for 30 min at 36 °C for 5 days a week. Pre- and posttreatment assessments of patients were performed, and samples were collected by a dermatologist. The findings revealed that in moderate psoriasis patients, a significant improvement in the PASI score was observed, and microbiome analysis revealed increased colonization of the *Leptolyngbya* genus and a decrease in the *Flavobacterium* genus. The microbiome colonization trend was the same for both psoriasis and nonpsoriatic skin area microbiome diversity. The *Leptolyngbya* genus is well known for its antibacterial, anti-inflammatory, and antioxidant activities, with an enrichment of lipids, carbohydrates, and proteins. *Leptolyngbya* also has tyrosinase inhibitory effects that could be responsible for the treatment of psoriasis (Ratnaparkhe et al. 2021).

The thermal water of Blue Lagoon also has antipsoriatic potential. The blue lagoon has several unique properties, such as the minerals and silica brine present in the blue lagoon, which are not present in any other sea. The silica brine present in the blue lagoon forms colloidal particles that further precipitate to form layers of white mud in the sea with a soft and soothing texture. This soft-textured silica mud is known to be effective for treating psoriasis lesions when it is applied to psoriasis lesions. Moreover, the unique species of algae *Leptolyngbya erebi* var. *Thermalis* dominate among other organisms in blue lagoons, with rapid growth in warm water, and are found nowhere else in other seas (Pétursdóttir & Kristjánsson 1996). In addition to short periods of summer, the sunlight period in Blue Lagoon, Iceland, is not favorable for the treatment of psoriasis.

Therefore, studies on the use of blue lagoon thermal water for the treatment of psoriasis are limited. Eysteinsdóttir et al. (2014) conducted a comparative nonrandomized clinical trial involving psoriasis patients for 6 weeks and demonstrated the beneficial effects of photobalanotherapy in the treatment of psoriasis. The intervention included 3 treatment regimens. In the first group, combined treatment (balneotherapy and NB-UVB) was given 3 times a week for 6 weeks; in the second group, daily combined treatment (balneotherapy and NB-UVB) was given daily for 6 weeks; and in the third group, only NB-UVB treatment was given 3 times a week for 6 weeks. Disease improvement was assessed by the psoriasis area severity index (PASI), epidermal thickness, histological changes (skin-homing chemokines CCL17, Th17, Tc17, T22, CD3 +, CD4 +/CD8 +, CLA), and quality of life (dermatology life quality index). The results revealed remarkable improvement in the PASI score (68.1% and 73.1%) in the first two groups of patients treated with balneophototherapy compared with the third treatment regimen. The quality of life and histological parameters also improved remarkably. Circulating Th17 (CD4 + CD45RO + IL23R + T cells) and Tc17 (CD8 + CD45RO + IL23R + T cells) cells were significantly decreased by more than 60%. Immunohistochemical analysis revealed a remarkable reduction in CD3^+^, CD4^+^, and CD8^+^ cells in the skin. The immunological biomarkers used in this study are controversial, as few studies have shown a correlation between these biomarkers and psoriasis activity. On the other hand, studies do not support this correlation. Similarly, the study reported by Eysteinsdottir et al. (2014) did not find a correlation between CD3^+^, CD4^+^, and CD8^+^ cells in psoriatic skin and the PASI score. Therefore, researchers are now more interested in identifying additional reliable immunological biomarkers other than IL-17 levels in the blood that have been used as promising biomarkers of psoriasis activity. With respect to the present literature on the therapeutic effects of blue lagoon on psoriasis, the literature is limited to identifying blue lagoon as a complementary approach for psoriasis treatment. The present study presents immunological biomarkers that represent controversy as to whether they are reliable biomarkers of psoriasis. Henceforth, further research is needed to identify biomarkers of psoriasis and the extent of treatment efficacy of thermal water using a large cohort of patients in clinical trials to reduce bias as well as in vitro studies to obtain a deep understanding of the mechanism of action of thermal water.

### Thermal water in Inherited ichthyosis management

Inherited ichthyosis is a congenital skin disease characterized by scaling, itching, xerosis, and inflammation of the skin. It has visible clinical symptoms, hence degrading quality of life. To date, there is no promising treatment other than symptomatic treatment involving the use of topical keratolytic agents, moisturizers, emollients, or retinoids to hydrate the skin and prevent itching and scaling or mechanical scale removal (Oji et al. 2010).

Thermal water could be effective in the treatment of ichthyosis. However, evidence on the treatment of ichthyosis is very scarce. Bodemer et al. (2011) first studied the effects of thermal water for the treatment of ichthyosis. For this intervention, 24 adults and 20 children with ichthyosis were enrolled for 18 days of hydrotherapy. The patients were supposed to perform follow-up visits 2 months before hydrotherapy and after 3 and 6 months of hydrotherapy for disease condition analysis by the same dermatologist. The assessment parameters used were the Dermatology Life Quality Index (DLQI) improvement score and ichthyosis severity, which were analyzed via the clinical ichthyosis score. The results suggested significant improvement in the DLQI at the end of the thermal water treatment and even 3 and 6 months after treatment. The other clinical symptoms also improved (Bodemer et al. 2011).

A case report study revealed complete clearance of the skin after 40 treatments of balneophototherapy in a patient with ichthyosis linerais circumflexa. The treatment modality included a saltwater bath in combination with UV-B radiation therapy 3–5 times a week for a total of 40 treatments. However, the disease relapsed after 4 months of treatment. Short-term disease relapse can be controlled by intermittent balneophototherapy (Gambichler et al. 2000).

In essence, the use of thermal water for the treatment of ichthyosis management has not been explored enough, and only a few studies have validated the partial beneficial effects of the availability of thermal water for ichthyosis treatment. In addition to the availability of thermal water, no other medicinal water has been investigated for its therapeutic potential in the treatment of ichthyosis. Future research should incorporate different types of thermal water in clinical, preclinical, and in vitro studies to investigate its therapeutic efficacy in treating ichthyosis.

### Thermal water in lichen planus management

Lichen planus is a chronic inflammatory disease affecting mostly the middle-aged population, with a 0.1–2.4% prevalence (Walton et al. 2010). The pathogenesis of lichen planus involves the upregulation of keratinocyte apoptosis and the low production of T cells, which ultimately leads to damage to the basal layer (Lei et al. 2010). The clinical treatment consists of corticosteroid administration. However, corticosteroids have many systematic adverse effects, and they cannot be used for a long duration. The treatment of lichen planus balneotherapy may be alternated or combined with traditional medication to achieve the best results.

Considering this, a clinical, open-label study evaluated the therapeutic effects of thermal water on lichen planus. The interventions included oral baths, vaporization, gingival showers, compresses, and hydropinotherapy (drinking 1.5 L of Avene thermal water) for 21 days. After treatment with thermal water, the symptoms of lichen planus (itching, erosion, erythema) significantly improved. Patients’ improvement was significant enough that they did not need any further topical corticosteroids (74%), analgesics (98%), or mouthwash corticosteroids (66%) (Boisnic et al. 2004). Even though beneficial effects of available thermal water have been observed for the treatment of lichen planus, the underlying mechanism is unclear.

### Thermal water in acne vulgaris management

Acne vulgaris is a chronic inflammatory disease of pilosebaceous ducts with a high prevalence in teenagers and is associated with puberty. Four major processes are involved in acne pathogenesis: inflammation, abnormal keratinocyte proliferation, increased sebum production, and increased production of *Propionibacterium acnes* (Gollnick et al. 2003; Thiboutot et al. 2009). Sulphuric thermal water, due to its keratolytic effect, is known to treat acne by reducing follicular obstruction (Soroka et al. 2008). Alirezai et al. (2000) conducted a multicenter, open-label comparative study with 69 acne patients after treatment to evaluate the effects of thermal water and retinoic acid for the treatment of facial acne. The treatment duration was 28 days, and after the treatment period, hey elucidated the marked effectiveness of thermal water combined with retinoic acid for the treatment of acne (desquamation) compared with that of only retinoic acid treatment.

Thermal water has the potential to cure acne via different pathways, such as follicular obstruction, sebum production regulation, reducing the viability of acne-causing Flavibacterium acne bacteria, and reducing inflammation. Henceforth, thermal water and balneotherapy can be effective complementary and well-tolerated modalities for acne treatment with no side effects.

## Cosmetic effects of thermal water

Cosmetic products can be categorized in different areas, such as color cosmetics, hair color, skincare, personal care products, and body care cosmetics. Cosmetics can be defined as any agent or mixture proposed to be applied externally to the human body to clean, protect, perfume, correct body odor, or change in appearance (EU Regulation 1223/2009, Article 2.1.a). Cosmetics are made up of many active substances along with some ancillary ingredients. Water is mostly used as an ancillary product in cosmetics, such as in lotions, emollients, creams, gels, etc. Hence, thermal water can be used in cosmetic formulations as an active and/or ancillary ingredient to enhance their beneficial properties.

Thermal water use for dermatological cosmetics has been studied in the context of skin regeneration, skin hydration, wound healing, acne treatment, and antiaging, antiwrinkling, soothing, desensitizing, and antioxidant effects on the skin. The thermal water-formulated cosmetic products are in the form of gels, lions, sprays, and creams (Araujo et al. 2017; Joly et al. 2014; Nunes et al. 2019) (Tables 3, 4). The cosmeceutical properties of thermal water are associated with its physiochemical composition as well as the microbial profile of the thermal water used in formulation (Almeida et al. 2019; Figueiredo et al. 2023; Joly et al. 2014).

Vendrely et al. (2022) developed an emollient plus balm using Avene spring thermal water extract of *Aquaphilus dolomiae* (ADE-G1) as the active ingredient, studied its effects on xerosis and tested it in patients receiving anticancer therapy. They reported good tolerance of the cream, even in patients receiving anticancer therapy, and that the severity of xerosis was markedly reduced. Similarly, in another study, the Avene thermal water *Aquaphilus dolomiae* extract formulated as an emollient was reported to have antixerotic, soothing effects in dermatological and systematic disease patients (Deleuran et al. 2020).

The antiaging effects of thermal water-derived algae extract (filamentous and coccoid algae) and silica mud-formulated cream are also described. The blue lagoon thermal water cream was shown to have antiaging effects through an increase in collagen 1 A1 and 1 A2 gene expression. The induction of involucrin, loricrin, transglutaminase-1 and filaggrin gene expression in primary human epidermal keratinocytes was shown to improve skin barrier functions (Grether‐Beck et al. 2008). A decrease in the number of α-melanocytes due to the skin lightening effects of blue lagoon algae extract-formulated cream has also been reported (Grether-Beck et al. 2022). Similar skin tightening and improved skin barrier function were also reported in another study in which the Vichy thermal water probiotic fraction was used (Gueniche et al. 2022b). The probiotic fractions of the *Vitreoscilla filiformis* extract and volcanic viscosity thermal water combination were tested in different proportions against human keratinocytes for skin barrier function and against blood mononuclear cells for immune defense. The results demonstrated the potential of the probiotic fraction to have skin barrier function via keratinocyte differentiation, significant biochemical defense via the activation of antimicrobial peptides, and cellular anti-inflammatory properties via the protection of Langerhans cells and the increase in the IL-10/IL-12 ratio in response to UV exposure (A Gueniche et al. 2022a, b).

The addition of Comano thermal water has been shown to promote both wound healing and skin regeneration (Faga et al. 2012; Nicoletti et al. 2019). Although there is a lack of explanation for the underlying mechanism by which Comano water accelerates the wound healing process, the high bicarbonate content of Comano water could play a role in increasing wound healing activities. BBS-rich water is known to enhance wound healing by increasing vessel density and reducing the number of inflammatory cells, retaining moisture and thermal insulation, and increasing the expression of matric metalloproteineases 2 and 9 (Liang et al. 2015).

Moreover, a clinical trial of 30 psoriasis and atopic dermatitis patients with different stages of disease for 15 days was carried out in which a cream composed of thermal water was used. The monfortitious water-based cream well described its skin hydrating effects by reducing the pruritic area and improving erythema in enrolled subjects in clinical trials. A clinical trial revealed improvements in erythema, itching, and skin flaking of 71%, 86%, and 86%, respectively, after treatment with monfortinho-based cream (Almeida et al. 2019).

### Thermal water anti-inflammatory and immunomodulatory effects

The applications of thermal water in the treatment of different inflammatory skin conditions have been well described in various clinical and preclinical studies. The thermal spring water mostly used for anti-inflammatory disease treatment is low to moderately mineralized and is mostly enriched in silica, selenium, magnesium, zinc, and some other minerals (Guerrero & Garrigue 2017; Nocera et al. 2020). Thermal water is known to inhibit TNF-alpha, E-selectin, and ICAM-1 expression, leading to the activation of the NF-κB transcription pathway (Castex-Rizzi et al. 2011). Thermal spring water has been reported to have the potential to reverse ROS formation, and IL-6 can be induced by exposure to UVB (Zoller et al. 2015).

The Avene thermal spring water demonstrated anti-inflammatory and immunomodulatory properties by inhibiting the expression of inflammatory mediators, thymic stromal lymphopoietin, interleukin (IL)−18, IL-4R, IL-8, IL-4, IIL-12, and IL-23 and activated innate immunity through toll-like receptor (TLR) 2, TLR4, and TLR5 activation (Aries et al. 2016; Eliasse et al. 2019). Portales et al. (2001) also studied the immunomodulatory effects of Avene thermal spring water on normal peripheral blood lymphocytes. The results revealed that significant inhibition of IL-4 production and modulation of the immunological Th2 profile in atopic dermatitis further promoted the lymphoproliferative response to some mitogens. IL-2 and IFN-γ production was increased. Similarly, Eliasse et al. (2020) studied Avene thermal water and demonstrated its modulatory effects on CD4 + T cells, dendritic cells (DCs), and mast cells. The anti-inflammatory activity of Comano thermal water has also been studied, and it has been reported to actively decrease inflammatory cytokines. Dal Pra et al. (2007) and A. Chiarini et al. (2006a, b) demonstrated the role of Comano thermal water in the significant inhibition of intracellular TNF-alpha, resulting in the downregulation of IL-8 and IL-6 in keratinocytes, which ultimately led to the control of the abnormal differentiation of keratinocytes, opening ways to treat skin diseases with underlying abnormal differentiation of keratinocytes. Dead Sea water is also known for its anti-inflammatory properties. Portugal-Cohen et al. (2011) reported remarkable inhibition of the inflammatory interleukins IL-6, IL-8, and IL-1α in human skin culture in response to UVB exposure in response to treatment with a thermal water-prepared cream. Similarly, in another study, the anti-inflammatory effects and antipollution effects on human-derived epidermal keratinocytes were studied against natural mineral thermal water and anionic polysaccharides (PolluStop®). Significant downregulation of prostaglandin and IL-1α production was observed (Portugal-Cohen et al. 2017). The high contents of magnesium, calcium, and selenium in the Dead Sea could be responsible for its anti-inflammatory properties. It has been proposed that magnesium inhibits TNF-a production, and zinc and calcium are known to bind inflammatory interleukins to suppress their production; in this way, they induce anti-inflammatory effects (Kim et al. 2010; Tarnowska et al. 2020).

La Roche Posay thermal water is a medium-sized mineral water with high concentrations of selenium, and it has also been revealed to have anti-inflammatory effects. Numerous in vitro studies (Celerier et al. 1995; Zoller et al. 2015) have reported the anti-inflammatory potential of La Roche Posay water using human-derived keratinocyte HaCT cells. The results revealed the suppression of proliferation, reversal of cell damage, and inhibition of the production of the inflammatory interleukin IL-6 in HaCT cells. The anti-inflammatory properties could be attributed to the high selenium levels of the Roche Posay water because selenium can modulate selenoprotein gene expression by inhibiting the nuclear factor NF-Kappa B and inflammatory interleukins (Duntas 2009).

Vichy thermal water is also known to have antioxidant defense activities, improving skin barrier function and strengthening immune defense (Rasmont et al. 2022). The protective effects of Vichy thermal spring water on Langerhen cells against UVB, antimicrobial peptide defense, and the downregulation of the interleukins IL-8, IL-23P40, and IL-12 have also been reported (Gueniche et al. 2022a).

## Thermal water antioxidant activities

Numerous studies have demonstrated the antioxidant activities of thermal water. The skin has different defense mechanisms against free radicals/reactive oxygen species. The most pronounced antioxidant defense comprises antioxidant enzymes (superoxide dismutase, catalase, glutathione peroxidase, glutathione reductase, etc.) and nonenzymatic antioxidants such as tocopherols, phenolic compounds, carotenoids, ascorbic acids, and Mycosporine-like amino acids (MAAs).

Tacheau et al. (2018) reported significant antioxidant effects of Vichy thermal spring water on human keratinocytes. They reported that DNA repair; increased expression of skin homeostasis-related genes; and the expression of antioxidant enzymes (TXNRD1, NQO1, GPx, CAT, and PRDX1, 2, 3, and 6) regulated by the Keap1‒Nrf2 pathway prevent oxidative stress-mediated skin aging.

Altaany et al. (2019) evaluated the effects of sulfurous thermal water on healthy subjects living in the vicinity of Al-Hammah sulfurous springs. The control group included residents of another region (Der-Allah region) but with the same sea level and geography. Blood samples were collected for serum analysis of total antioxidant capacity (TAC), the oxidative stress index (OSI), and total oxidative stress (TOS). The results revealed significantly greater TAC and lower OSI and TOS values in the experimental group than in the control group.

Similarly, the antioxidant effects of another profoundly highly mineralized water, Saturnia sulfurous spring water, have been reported. Benedetti et al. (2009) reported a significant increase in antioxidant levels in subjects treated with Saturnia thermal for 2 weeks. Lipid peroxidation protein oxidation and advanced oxidation products were significantly lower in the sulfurous thermal water-treated group than in the control group, and the antioxidant capacity was also improved. The mechanism behind the potent antioxidant effects of this sulfurous thermal water might be the high penetration power of sulfur in cells and its diverse ability to react with and eliminate 4 types of ROS: superoxide radicals (Mitsuhashi et al. 2005), peroxynitrite (Whiteman et al. 2005), hypochlorite (Whiteman et al. 2006), and hydrogen peroxide (Geng et al. 2004). It also has radical scavenging potential (Kimura & Kimura 2004). Therefore, sulfur from thermal water could be an influential element in regulating oxidative stress and increasing antioxidant activities.

## Mechanism of action of thermal water

Balneotherapy has been shown to effectively treat dermatologic conditions and play role as cosmetic effects. While the precise mechanisms by which thermal waters provide such a wide range of benefits is still unclear. It is known that balneotherapy triggers neuroendocrine and immune responses, which contribute to its healing properties through anti-inflammatory, analgesic, antioxidant, chondroprotective, and anabolic effects, as well as neuroendocrine-immune regulation. Depending on their mineral composition, certain thermal waters may have specific therapeutic actions targeting various skin diseases.

Exposure to thermal waters activates the immune and antioxidant systems of the body. The components of thermal water such as magnesium, sulfur, bicarbonates, selenium and other salts play major role in its therapeutic action to treat different skin diseases (Joly et al. 2000; Takahashi et al. 2008). Magnesium from thermal water can improve polyamines production that are known to be responsible for psoriasis progression (Matz et al. 2003). Magnesium and zinc strengthen the skin barrier and support the immune system, while the combination of magnesium and calcium salts accelerates skin healing. An in vitro study has demonstrated that selenium and strontium can reduce cytokine production, particularly IL-6, in epidermal cells. Additionally, selenium has been shown to suppress the inflammatory response of Langerhans cells (LCs)(Wollenberg et al. 1992). As for silica and calcium bicarbonate, they are known to inhibit mast cell histamine release and reduce cutaneous basophil degranulation, which may help prevent the itch–scratch cycle associated with atopic dermatitis (Joly et al. 2000). Similarly, the sulfur from water can be implied as most predominant components for skin health mainly to treat chronic inflammatory skin diseases attributed to its antiproliferative, keratolytic, antioxidant, antifungal and anti-inflammatory properties. Sulfur from sulfurous water can effectively suppress production of inflammatory cytokines ((IL)−2, IL-8, IL-23, IL-17) and T lymphocytes proliferation (Lee et al. 2014; Ninković-Baroš et al. 2014). Sulfurous water balneotherapy is also validated to impede production of intercellular adhesion molecule 1 expression as well as E selectin in human endothelial cells therefore decreasing the disease progress (Lee et al. 2016). Some in vitro studies of sulfurous water provide evidence of immunomodulation of immune system. Moreover, sulfurous water is also reported to inhibit psoriasis inflammatory mediators such as decrease in production of il-2, il-6, interferon gamma, tumor necrosis factor-alpha (TNFα) and T lymphocytes proliferation (Castex‐Rizzi et al. 2011; Péter et al. 2017). The other way of action of sulfur from thermal water is to reduce the adhesion of leuckocytes and production of cytokine in skin layers and level up production of β-endorphins. Moreover, thermal water rich in salts improve blood flow, regulate elastase enzyme production, dilate capillaries, and lower fibrinogen levels, potentially easing skin diseases traits by reducing inflammation and promoting skin healing, increasing patients’ quality of life (Brockow et al. 2007).

On the other hand, the thermal effect of thermal water also play major role in treating skin diseases by increasing production of cortisol, β-endorphins, prolactin, norephinephrine, adrenocorticotropic hormone and prolactin and also decrease basophils degranulation in patients of atopic dermatitis (Borroni et al. 2013; Sacerdote et al. 2002). Treatment with thermal water at a specific temperature, through thermal stimulation, induces vasodilation, improves blood circulation, and lowers blood pressure by regulating elastase enzyme production in various skin layers, dilating capillaries, and lowering fibrinogen levels, which may help reduce psoriasis complications (Brockow et al. 2007). Hyperthermia also significantly influences granulocyte mobility, as well as microbial and enzymatic activities. Additionally, thermal stimulation enhances the extensibility of collagen-rich tissues beneficial for skin health (Bogdanov et al. 2012; Golušin et al. 2015). In conclusion, the physical and chemical properties of thermal waters play a crucial role in their therapeutic effects, and careful selection is essential when recommending them for the treatment of various dermatological conditions.

## Spatial and temporal trend of balneotherapy studies

**Fig. 4:**
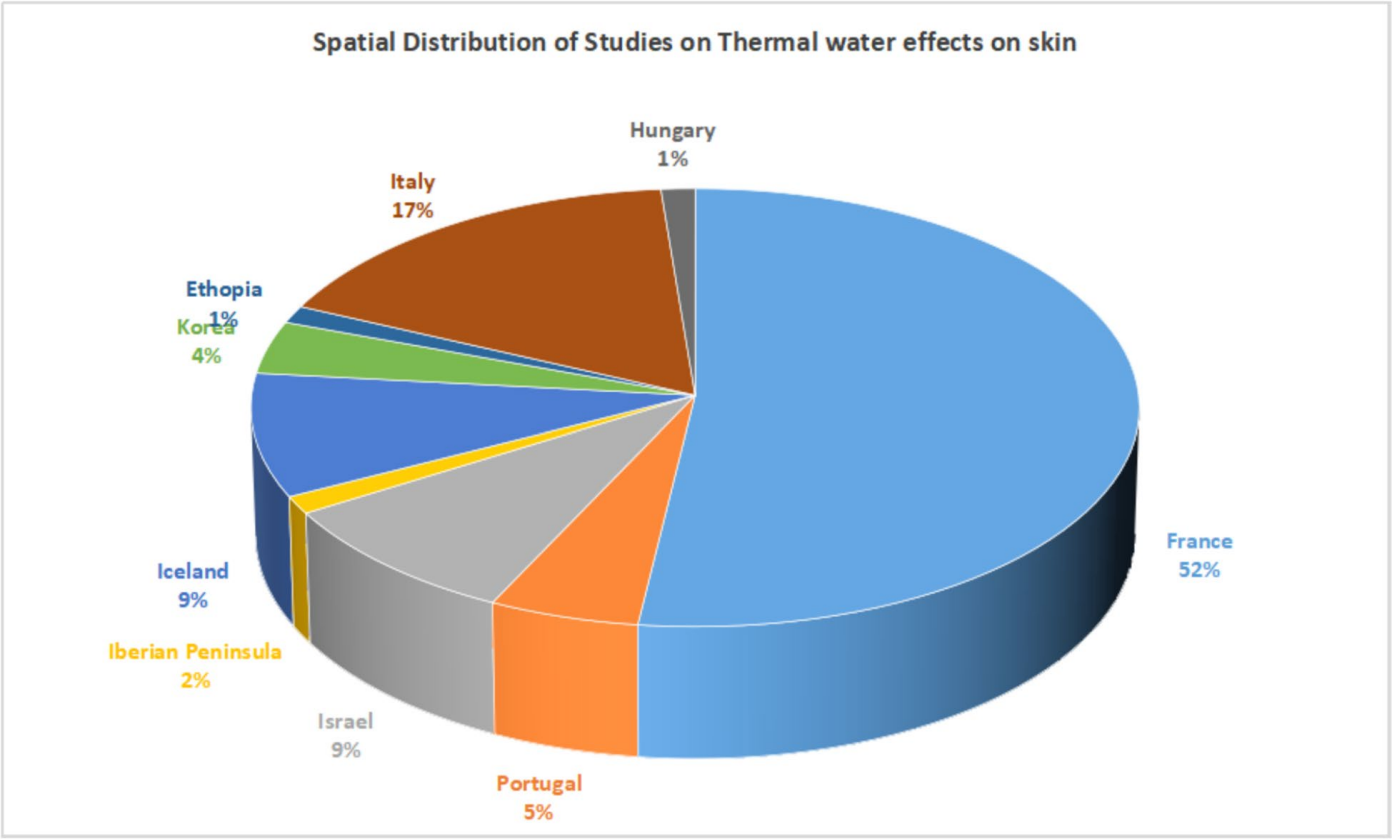
The spatial distribution of in vitro and in vivo studies on thermal water entailing its therapeutic and cosmetic effects

The global distribution of studies on thermal water balneotherapy for skin diseases and cosmetic effects reveals a notable attention in specific regions (Fig. 4). France stands out as a dominant contributor, with a significant number of studies, especially concentrated on well-known thermal springs like Vichy, La Roche-Posay, and Avene."Italy follows with a notable presence of studies centered around thermal springs like Comano and Saturnia, while other Italian regions such as Levico and Vetriol contribute to the research landscape. The Dead Sea in Israel and the Blue Lagoon in Iceland are also prominent sites of investigation. Other countries like South Korea, Hungary, Portugal, and Ethiopia are represented by a limited number of studies, often focusing on specific springs such as the Dead Sea, Deokgu, and Lake Hervaiz.However, several regions, including large parts of Africa, South America, and Asia (excluding the Middle East and Korea), remain largely unexplored in the context of thermal water and its effects on skin diseases and cosmetic outcomes. This spatial gap suggests a potential area for further research, particularly in underrepresented continents such as Africa and South America, where the therapeutic properties of local thermal waters remain largely undocumented.

**Fig. 5:**
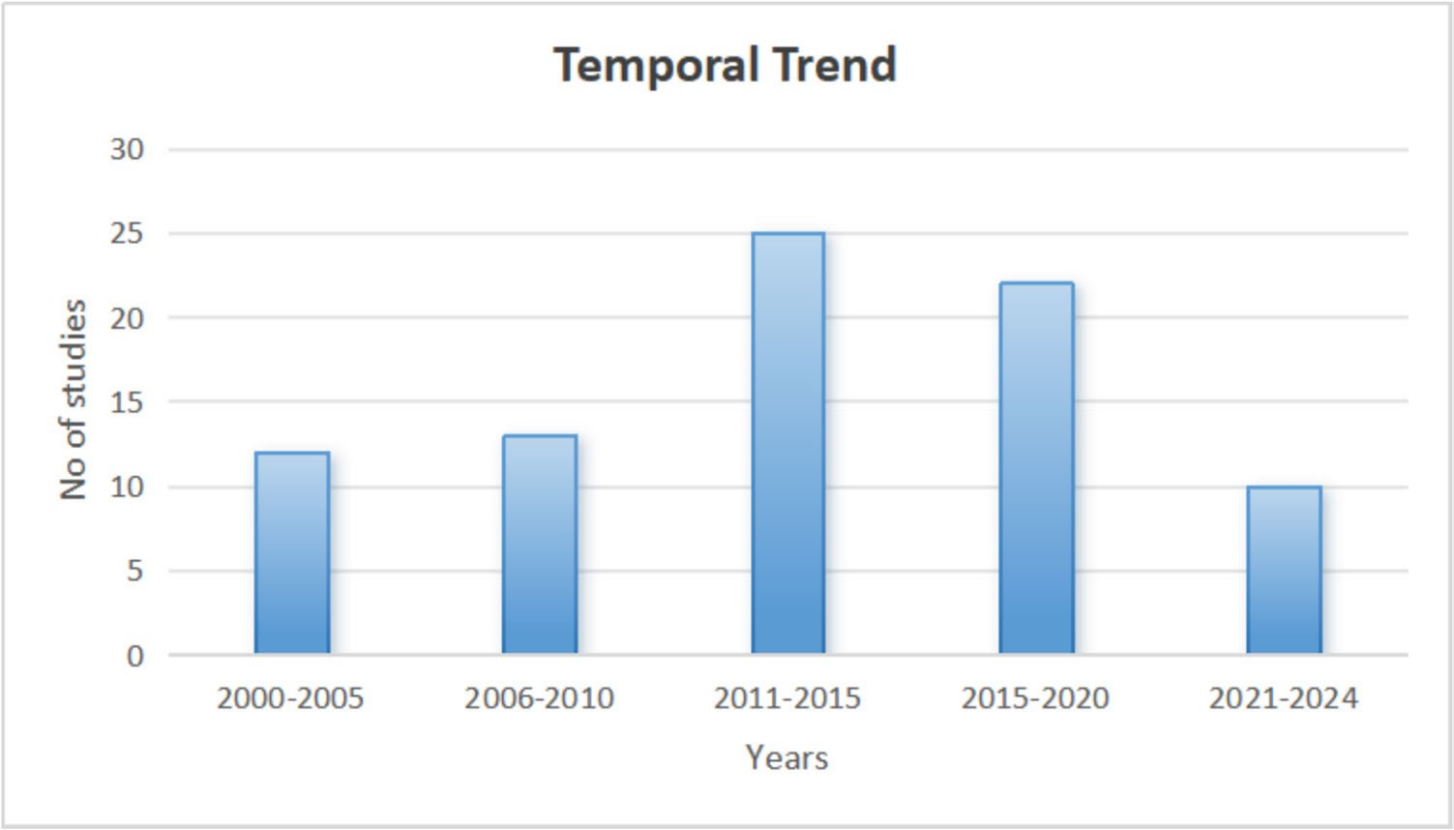
The temporal trend of literature on therapeutic and cosmetic effects of thermal water from 2000–2024

In terms of temporal trends, the number of studies has steadily increased over the past two decades, with a noticeable rise in research activity from 2000 to 2015 (Fig. 5). From 2000 to 2005, only 12 studies were conducted, which grew to 13 between 2006 and 2010. The period from 2011 to 2015 observed more significant increase, with 25 studies published. However, research activity slightly declined from 2015 to 2020, with 22 studies, and further decreased to 10 studies between 2021 and 2024. This suggests that while interest in thermal water balneotherapy was peaking in the early 2010 s, the momentum has slightly waned in recent years. The combination of spatial and temporal trends underscores the need for further research, particularly in underrepresented regions, and suggests the potential for a resurgence of interest in thermal water studies in the coming years.

## Future research perspective

Generally, the literature validates the potential of thermal water treatment, balneotherapy, as an effective strategy in the treatment of skin diseases, as well as its cosmetic potential. However, there is research gaps in the context of specific types of thermal water and its therapeutic and dermo-cosmetic effects. For example, there is no clear view available demonstrating the underlying mechanism of action of different types of thermal water and the treatment of certain skin diseases or their cosmetic effects. Therefore, future studies should focus on the molecular and cellular mechanisms elicited by various types of thermal water, using various matrices, such as primary cell cultures, tissue explants obtained from diseased skin, or complex cellular models, such as 3D models of primary cell culture, to investigate the effects of specific types of thermal water for the treatment of certain skin diseases. To date, research on thermal water has focused on psoriasis and atopic dermatitis, with a limited focus on ichthyosis, acne, vitiligo, melanoma and basal cell carcinoma. Although advancements in drug delivery have offered many advantages and increased the effectiveness of targeted drug delivery, this advanced drug delivery approach has been completely neglected with respect to the medicinal effects of thermal water. Compared with the physiochemical properties of thermal water, less attention has been given to the microbiome of thermal water. Furthermore, there is debate over the efficacy and reliability of in vivo studies of balneotherapy, such as in vivo studies that provide only short-term effects of balneotherapy with no information on long-term effects. There is no evidence of the absorption of minerals from thermal water into the circulatory system. Owing to the heterogeneity of thermal water composition from different areas and differences in the duration of treatment, it is very difficult to compare the results of balneotherapy and derive optimal clinical and biological outcomes. To overcome such bias, in the future, in vivo and in vitro studies should be performed to obtain a better understanding of the mechanism of action of thermal water and skin disease pathology.

## Conclusion

This article provides a detailed summary of various types of thermal spring water used for skin disease treatment as well as their cosmetic effects on the skin. This comprehensive information highlights the significant therapeutic and cosmeceutical potential of thermal water for the treatment of different skin diseases (successfully treated skin diseases, including psoriasis, atopic dermatitis, and xerosis) and for enhancing skin properties, such as skin hydration, antiaging, skin regeneration, and wound healing effects. Thermal water has also been shown to have anti-inflammatory, immunomodulatory, anticarcinogenic, and antioxidant properties. Although thermal water has been well known for its tremendous benefits in manifestations of skin diseases and cosmetic effects, the underlying mechanism behind its miraculous properties is unclear. This limitation prevents the full unlocking of thermal water use in medical forums to extended levels. Among the most remarkable medicinal waters, sulfurous thermal water is the most effective in terms of skin disease treatment.

## Data Availability

There are no data available for this manuscript.
